# The structural basis for release-factor activation during translation termination revealed by time-resolved cryogenic electron microscopy

**DOI:** 10.1038/s41467-019-10608-z

**Published:** 2019-06-12

**Authors:** Ziao Fu, Gabriele Indrisiunaite, Sandip Kaledhonkar, Binita Shah, Ming Sun, Bo Chen, Robert A. Grassucci, Måns Ehrenberg, Joachim Frank

**Affiliations:** 1 Department of Biochemistry and Molecular Biophysics, Columbia University, New York, NY 10032 USA; 2 Department of Cell and Molecular Biology, Uppsala University, Uppsala, 751 24 Sweden; 3 Department of Biological Sciences, Barnard College, New York, NY 10027 USA; 4 Department of Biological Sciences, Columbia University, New York, NY 10027 USA

**Keywords:** Single-molecule biophysics, Cryoelectron microscopy

## Abstract

When the ribosome encounters a stop codon, it recruits a release factor (RF) to hydrolyze the ester bond between the peptide chain and tRNA. RFs have structural motifs that recognize stop codons in the decoding center and a GGQ motif for induction of hydrolysis in the peptidyl transfer center 70 Å away. Surprisingly, free RF2 is compact, with only 20 Å between its codon-reading and GGQ motifs. Cryo-EM showed that ribosome-bound RFs have extended structures, suggesting that RFs are compact when entering the ribosome and then extend their structures upon stop codon recognition. Here we use time-resolved cryo-EM to visualize transient compact forms of RF1 and RF2 at 3.5 and 4 Å resolution, respectively, in the codon-recognizing ribosome complex on the native pathway. About 25% of complexes have RFs in the compact state at 24 ms reaction time, and within 60 ms virtually all ribosome-bound RFs are transformed to their extended forms.

## Abstract (web summary)

Translation termination is under strong selection pressure for high speed and accuracy. Here the authors provide a 3D view of the dynamics of a translating bacterial ribosome as it recruits a class-1 release factor (RF1 or RF2) upon encountering a stop codon, and propose a structure-based kinetic model for the early steps in bacterial translation termination.

## Introduction

Most intracellular functions are carried out by proteins, assembled as chains of peptide-bond linked amino acid (aa) residues on large ribonucleoprotein particles called ribosomes. The aa-sequences are specified by information stored as deoxyribonucleic acid (DNA) sequences in the genome and transcribed into sequences of messenger RNAs (mRNAs). The mRNAs are translated into aa-sequences with the help of transfer RNAs (tRNAs) reading any of their 61 aa-encoding ribonucleotide triplets (codons). In termination of translation, the complete protein is released from the ribosome by a class-1 release factor (RF) recognizing one of the universal stop codons (UAA, UAG, and UGA), signaling the end of the amino acid encoding open reading frame (ORF) of the mRNA. There are two RFs in bacteria, RF1 and RF2, one in eukarya, eRF1. RF1 and RF2 read UAA, UAG, and UAA, UGA, respectively, while the omnipotent eRF1 reads all three stop codons. Each stop codon in the decoding center (DC) is recognized by a stop-codon recognition (SCR) motif in a class-1 RF, and all RFs have a peptidyl transfer center (PTC)-binding GGQ motif, named after its universal Gly–Gly–Gln triplet (GGQ), for coordinated ester bond hydrolysis in the P-site bound peptidyl-tRNA. The crystal structures of free RF1 and RF2 have a distance between the SCR and GGQ motifs of about 20 Å^1,2^, much shorter than the 70 Å separating DC and PTC in the bacterial 70S ribosome. This distance discrepancy made the expected coordination between SCR and ester bond hydrolysis enigmatic. The crystal structure of free eRF1 has, in contrast, about 70 Å between its SCR and GGQ motifs, a distance close to the 80 Å between the DC and PTC of the 80S ribosome in eukarya^3^. Further cryo-EM work showed that ribosome-bound RF1 and RF2 have extended structures^4,5^, facilitating coordinated codon recognition in DC and ester bond hydrolysis in PTC. Subsequent high-resolution X-ray crystal^6–13^ and cryo-EM^14–19^ structures of RF-bound 70S ribosomes allowed the modeling of stop-codon recognition by RF1, RF2^20^, eRF1^21^, and GGQ-mediated ester bond hydrolysis^22^.

If the compact forms of free RFs in the crystal^1,2^ are physiologically relevant, it would mean that eubacterial RFs are in the compact form upon A-site entry (pre-accommodation state) and assume the extended form (accommodation state) in a stop-codon dependent manner. The relevance is indicated by a compact crystal structure of RF1 in a functional complex with its GGQ-modifying methyltransferase^23,24^, although SAXS data indicated free RF1 to be extended in bulk solution^25^. At the same time, SAXS data from T. thermophilus RF2 free in solution suggested a compact form for the factor or, possibly a mixture of compact and extended forms^26^. The existence of a RF-switch from a compact, free form to an extended ribosome-bound from would make high-resolution structures of these RF-forms necessary for a correct description of the stop-codon recognition process, hitherto based on post-termination ribosomal complexes^27,28^.

Indirect evidence for rapid conformational activation of RF1 and RF2 after A-site binding has been provided by quench-flow based kinetics^22^, and in a series of recent FRET experiments Joseph and collaborators showed free RF1 to be in a compact form^29^, compatible with the crystal forms of RF1^1^ and RF2^2^, but in an extended form when bound to the A site of the stop-codon programmed ribosome^29^. Ribosome-bound class-1 RFs in the compact form has been observed together with alternative ribosome-rescue factor A (ArfA) in ribosomal rescue complexes, which lack any codon in the A site^14,15^. Very recently, Svidritskiy and Korostelev^6^ used X-ray crystallography in conjunction with the peptidyl transfer-inhibiting antibiotic blasticidin S (BlaS) to capture a mutated, hyper-accurate variant of RF1 in the stop codon-programmed termination complex. They found RF1 in a compact form, which they used to discuss stop-codon recognition in conjunction with large conformational changes of the RFs. It seems, however, that this BlaS-halted ribosomal complex is in a post-recognition state (i.e., stop-codon recognition motif has the same conformation as in the post-accommodation state in DC) but before RF-accommodation in the A site, making its relevance for on-pathway stop-codon recognition unclear (However, see also below!).

Here, in contrast, we use time-resolved cryo-EM^30–34^ for real-time monitoring of how RF1 and RF2 ensembles change from compact to extended RF conformation in the first 100 ms after RF-binding to the pre-termination ribosome. These compact RF-structures, originating from short-lived ribosomal complexes previously out of reach for structural analysis, are seen at near-atomic resolution (3.5–4 Å). The time-dependent ensemble changes agree qualitatively with accompanying and previous^22^ quench-flow studies. We discuss the role of the compact structures of RF1 and RF2 for fast and accurate stop-codon recognition in translation termination.

## Results

### Kinetics study predicts compact RF1/RF2 exist at 20 ms

**Fig. 1:**
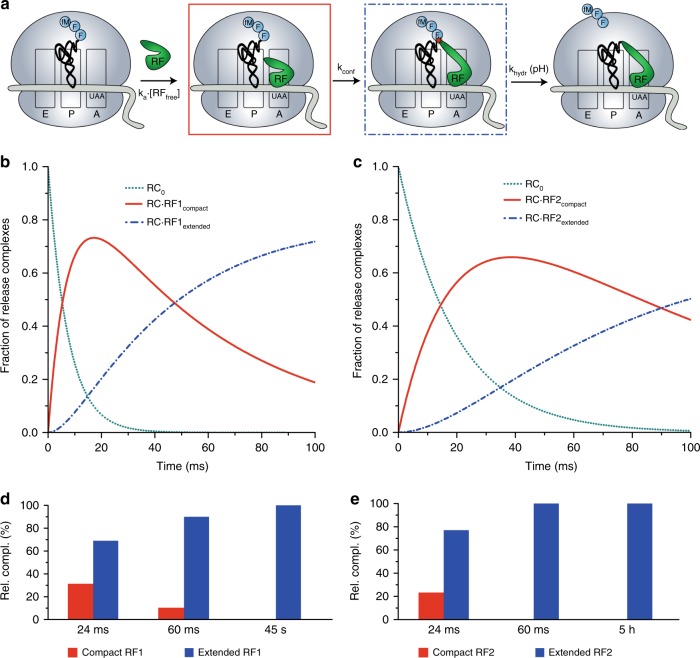
Time evolution of ribosome ensembles in termination of translation **a**. Cartoon visualization of the pathway from free release complex to peptide release. Compact class 1 release factor (RF) binds to RF-free ribosomal release complex (RC_0_) and forms the RC·RF_compact_ complex with compounded rate constant k_a_·[RF_free_]. Stop codon recognition induces conformational change in the RF which brings the ribosome from the RC·RF_compact_ to the RC·RF_extended_ complex with rate constant k_conf_. The ester bond between the peptide and the P-site tRNA is hydrolyzed with rate constant k_hydr_. **b** Predicted dynamics of peptide release with conformational change in RF1. We solved the ordinary differential equations associated with termination according to the scheme in **a** with association rate constant k_a_ = 45 µM^−1^s^−1^, [RF1_free_] = 3 µM, k_conf_ = 18 s^−1^ and k_hydr_ = 2 s^−1^ (Supplementary Fig. 2) and plotted the fractions of ribosomes in RC_0_, RC·RF_compact_ and RC·RF_extended_ forms (*y*-axis) as functions of time (*x*-axis). Green dot lines, RC_0_; red solid lines, RC·RF_compact_; blue dash lines, RC·RF_extended_. **c** Predicted dynamics of peptide release with conformational change in RF2. The fractions of ribosomes in different release complexes were obtained in the same way as **b** with the rate constants k_a_ = 17 µM^−1^ s^−1^, [RF2_free_] = 3 µM, k_conf_ = 11 s^−1^ and k_hydr_ = 2.7 s^−1^ (Supplementary Fig. 3). **d**, **e** The populations of release complexes containing compact conformation and extended conformation of RF1 (**d**) and RF2 (**e**) at the 24 ms, 60 ms and long incubation time points as obtained by time-resolved cryo-EM after 3D classification of the particle images

We assembled a UAA-programmed release complex, RC_0_, with tripeptidyl-tRNA in the P site, and visualized its structure with cryo-EM (Methods and Supplementary Fig. 1). The RC_0_ displays no intersubunit rotation, and the tripeptide of its P-site tRNA is seen near the end of the peptide exit tunnel (Supplementary Fig. 1). The mRNA of the DC is disordered, but the overall resolution of the RC_0_ is high (2.9 Å). Apart from a small fraction of isolated ribosomal 50S subunits, the RC_0_ ensemble is homogeneous (Supplementary Fig. 1). We used quench-flow techniques to monitor the time evolution of the class-1 RF-dependent release of tripeptide from the peptidyl-tRNA with UAA-codon in the A site after rapid mixing of RC_0_ with RF1 or RF2 at rate-saturating concentrations (k_cat_-range)^22^ (Fig. 1a). The experiments were performed at pH values from 6 to 8 units, corresponding to [OH^−^] variation in the 0.25–2.5 µM range (Supplementary Figs 2 and  3). The results are consistent with the existence of a two-step mechanism, in which a pH-independent conformational change (rate constant k_conf_) is followed by pH-dependent ester bond hydrolysis (see Methods). We estimate k_conf_ as 18 ± 3 s^−1^ for RF1 and 11 ± 1 s^−1^ for RF2 at 25 °C, which approximates the effective incubation temperature for the time-resolved cryo-EM experiments (Supplementary Figs 2 and  3).

From the quench-flow data, we predicted that the ensemble fraction of the compact RF1/2 form would be predominant at 24 ms, and much smaller at 60 ms (Fig. 1b, c). These predictions are in qualitative agreement with the time-resolved cryo-EM data, which show a somewhat faster conformational transition than in the quench-flow experiments (Fig. 1d, e). The difference in termination rates is, we suggest, due to a local temperature increase by friction inside the microfluidic chip. We first focus on the cryo-EM structures of RF1, and then highlight the few structural differences between RF1 and RF2.

### Time-resolved cryo-EM analysis

**Fig. 2:**
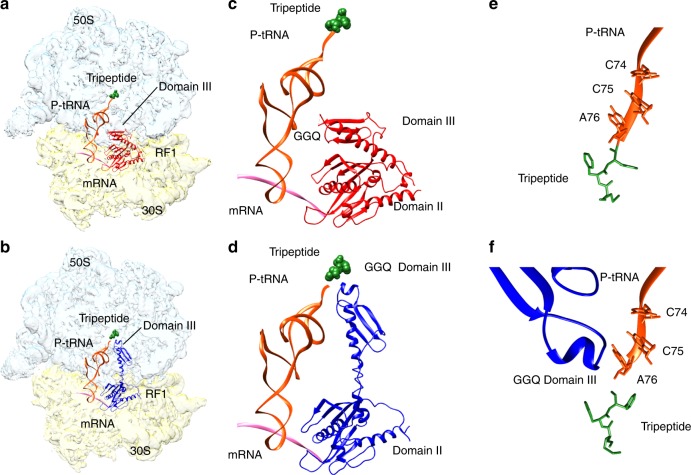
Cryo-EM structures of E.coli 70S ribosome bound with release factor 1. **a** Pre-accommodated ribosome complex bound with RF1 in a compact conformation. **b** Accommodated ribosome complex bound with RF1 in an extended conformation. Light blue: 50S large subunit; light gold: 30S small subunit; green: tripeptide; orange: P-tRNA; pink: mRNA; red: compact RF1; and dark blue: extended RF1. **c**, **d** Positions of domain III of ribosome-bound RF1 in pre-accommodated ribosome complex (**c**) and accommodated RF1-ribosome complex (**d**) relative to mRNA (pink), P-tRNA (orange) and tripeptide (green). **e**, **f** Close-up views of the upper peptide exit tunnel, showing tripeptide (green) in pre-accommodated ribosome complex (**e**) and accommodated ribosome complex (**f**)

At 24 ms reaction time, 25% of ribosome-bound RF1 is in the compact form in what we name the pre-accommodation state of the ribosome (Fig. 1d). The 70S part of the complex is similar to that of the pre-termination complex preceding RF-binding, but there is an additional A-site density belonging to RF1 (Fig. 2a). In pre-accommodation state of the ribosome, domain III of RF1 is 60–70 Å away from the PTC, in a similar relative orientation as in the crystal forms of the free factors^1,2^ (Supplementary Fig. 4) and significantly differing from that in the post-accommodated state of the terminating ribosome^5^. The loop that contains the GGQ motif of RF1 is positioned at the side of the β-sheet of domain II (near aa 165–168), facing the anticodon-stem loop and the D stem of the P-site tRNA (Fig. 2a, c).

At 60 ms reaction time the RF1-bound ribosome ensemble is dominated by the extended form of RF1 (Fig. 2b, d). We term the ribosome complex with extended RF1 the accommodated RF1-ribosome complex. It contains density for the tripeptide in the exit tunnel, indicating that at 60 ms the peptide has not been released from the ribosome (Fig. 2e, f and Supplementary Fig. 5). At a much later time-point (45 s) the tripeptide density is no longer present in the exit tunnel of the accommodated RF-ribosome complex. Precise estimation of the time evolution of tripeptide dissociation from the ribosome would require additional time points. Of particular functional relevance would be estimates of the time of dissociation of longer peptide chains from the exit tunnel.

The most striking difference between the compact and extended conformation of ribosome-bound RF1 is the position of the GGQ of domain III. As RF1 switches its conformation from the compact to the extended form, the repositioning of domain III places the catalytic GGQ motif within the PTC, and adjacent to the CCA end of the P-site tRNA (Fig. 2c, d). The extended form of RF1 has a similar conformation as found in the previous studies^7,10,12,13,35,36^.

**Fig. 3:**
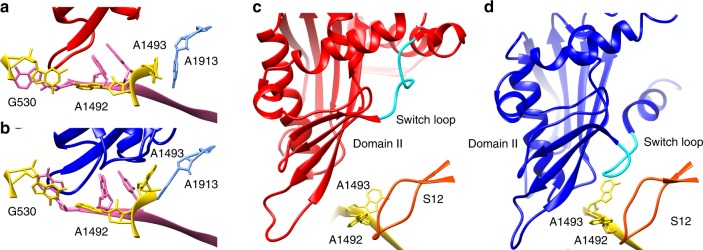
Interaction of RF1 with the ribosomal decoding center. **a**, **b** Structures of the ribosomal decoding center in pre-accommodated ribosome complex (**a**) and accommodated ribosome complex (**b**). Red: compact RF1; dark blue: extended RF1. **c**, **d** Conformations of switch loop in pre-accommodated ribosome complex (**c**) and accommodated ribosome complex (**d**). Gold: A1492 and A1493; and orange: S12

Similar to the case of sense-codon recognition by tRNA, three universally conserved DC residues, A1492, A1493, and G530 of the ribosome’s 16S rRNA undergo key structural rearrangements during translation termination. In the RF-lacking termination complex, A1492 of helix 44 in 16S rRNA stacks with A1913 of H69. A1493 is flipped out and stabilizes the first two bases in the A-site stop codon. G530 stacks with the third base A in the stop codon. In the presence of RF, whether compact or extended, A1492 is flipped out towards G530 and interacts with the first two stop-codon bases. A1493 stacks with A1913, which is in close contact with A1492 in the RF-lacking termination complex. G530 stacks with the third stop-codon base (Fig. 3a, b).

The switch loop, which was previously proposed to be involved in inducing a conformational change of RF1^10,36^, shows no interaction with protein S12 or 16S rRNA in the compact form of RF1 (Fig. 3c) whereas in the extended form of RF1, the rearranged conformation of the switch loop is stabilized by interactions within a pocket formed by protein S12, the loop of 16S rRNA, the β-sheet of domain II, and A1493 and A1913 (Fig. 3b, d). Shortening the switch loop (302–304) resulted in a substantially slower, rate-limiting step in peptide release^37^, which indicates that the switch loop plays a role in triggering the conformational change of RF1.

Similar experiments were carried out for RF2 at 24 ms, 60 ms and 5 h reaction times. RF2 undergoes a conformational change from compact to expanded form similar to that of RF1 (Fig. 1e). As in the case of RF1, the switch loop of RF2 makes no contact with protein S12 or 16S rRNA. In the extended form of RF2, A1492 is flipped out from helix 44 (h44) of 16S rRNA and stacks on the conserved Trp319 of the switch loop, stabilizing the extended conformation of RF2 on the ribosome.

The ribosome complexes with RF1/2 bound in compact conformation seen here are distinct from those reported for the ribosome rescue complex, in which ArfA is bound in the A site lacking a stop codon^14,15^. In our structures, the conformation of the conserved 16S rRNA residues in the DC (A1492, A1493, and G530) is similar to the classical termination configuration^10^. In contrast, in the presence of ArfA, these residues adopt conformations known from sense-codon recognition^14,15^. It suggests that the compact RFs bind to the A site regardless of the conformation of the DC. The conformational change of RFs is likely due to the changes in the switch loop triggered by its interaction with protein S12 and 16S rRNA. This interaction is disrupted by the mutation A18T of ArfA, hence leaving RFs in the compact conformation^15^.

Our ribosome complexes with RF1/2 are also distinct from a recent ribosome complex with compact RF1, reported by Svidritskiy and Korostelev^6^. Shortening of the switch loop, combined with the addition of the antibiotic BlaS which prevents the GGQ motif from reaching the PTC, stabilizes ribosome-bound RF1 in a compact conformation^6^, distinct from the transient, compact RF1-structure observed here. In our structure, the SCR between the β4–β5 strands on domain II are bound loosely to the A site (Fig. 3a). In the BlaS-halted compact RF1 structure^6^, in contrast, the stop codon-recognition motif of RF1 has moved further into the A site by 5 Å, to a position almost identical to that of the fully accommodated, extended structure of RF1. The functional role of their structure is not immediately obvious, but if it can be interpreted as an authentic transition state analogue, the roles of our respective complexes would be complementary. We previously found that high accuracy of stop signal recognition depends on smaller dissociation constant (K_m_-effect) and larger catalytic rate constant (k_cat_-effect) for class-1 RF reading of cognate stop codons compared to near-cognate sense codons^38^. The K_m_-effect contributes by factors from 100 to 3000 and the k_cat_-effect by factors from 2 to 3000 to the overall termination accuracy values in the 10^3^–10^6^ range^38^. Accordingly, the present structure may represent binding of RFs in a transient state where rapid and codon-selective dissociation rates are responsible for the accuracy factor due to the K_m_-effect. Furthermore, Korostelev's structure^6^, with its comparatively deep interaction between the cognate stop codon and SCR center, could mimic the authentic transition state on the path from compact to the extended form of the RF. Accordingly, Korostelev's structure may illustrate additional selectivity due to the k_cat_-effect. To test these hypotheses, molecular computations^28^ based on our respective RF structures could be used to compare their stop codon selectivities with those of RFs in the post-termination state of the ribosome^20^.

In a recent paper on the role of RF3 in the dissociation of the release factors RF1 and RF2^39^, the authors observed an interaction between domain I of RF1 and L7/L12 proteins, which assists the binding of RF1, as supported by complementary functional analysis using L7/L12 deletion mutants. However, such an interaction is not observed in our structures. Another recently published study using smFRET^40^ reported two states of the termination complex, non-rotated and rotated, in apparent contradiction to our results as we only found one, non-rotated state. The rotated-state subpopulation observed by Adio et al.^40^ may represent the post-termination ribosome unbound to RF1/RF2, as also suggested by previous single-molecule work from Puglisi and Gonzalez labs^41,42^.

## Discussion

**Fig. 4:**
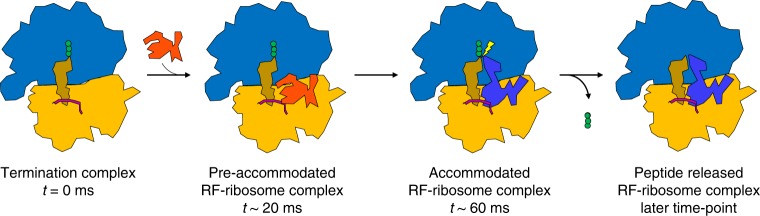
Structure-based model. The sequence of states is (1) the termination complex with the stop codon at the A site, (2) the initial binding state (RF compact; “pre-accommodated RF-ribosome complex”), (3) the open catalytic state (RF open/extended; “accommodated RF-ribosome complex”) and (4) the state after peptide release. (The later time point is not known from our experiment, and we only know from another experiment that the final state was seen after 45 s.) Blue: 50S large subunit; orange: 30S small subunit; green: tripeptide; brown: P-tRNA; pink: mRNA; red: compact RF; and blue-purple: extended RF

During translation termination, the release of the nascent peptide must be strictly coordinated with the recognition of a stop codon at the A site. Our cryo-EM analysis shows that in the presence of a class-1 RF the bacterial ribosome adopts several states. After rapid addition of RF1 or RF2 to a ribosomal termination complex with tripeptidyl-tRNA attached at the P site, we first observe the pre-accommodated RF-ribosome complex (compact form of RF) at 24 ms with the peptide attached to the P-site tRNA. This, we suggest, is the first step in the termination reaction. Second, at 60 ms, we observe the accommodated RF-ribosome complex with the extended form of RF and the tripeptide in the exit tunnel. Third, at a much later time point, we observe the post-accommodated RF-ribosome complex, with the extended form of RF without tripeptide in the exit tunnel (Supplementary Fig. 5). These pieces of evidence from our time-resolved experiments clearly reflect the sequence of events in termination of bacterial protein synthesis. A structure-based model for the stepwise interaction between ribosome and RF and the release of the nascent peptide from the termination complex during the translation termination process is presented in Fig. 4. It shows how the ribosome traverses (1) the pre-termination state with the stop codon at the A site, (2) the initial binding state (RF compact; pre-accommodated RF-ribosome complex), (3) the open catalytic state (RF open/extended; accommodated RF-ribosome complex) and (4) the state after peptide release. We suggest that the selective advantage of the compact RF-form is that it allows for rapid factor binding into and dissociation from an accuracy maximizing pre-accommodation state.

## Methods

### Components for in vitro translation and fast kinetics

Buffers and all *Escherichia coli* (*E. coli*) components for cell-free protein synthesis were prepared as described^22^. Ribosomal release complexes (RC) contained tritium (^3^H) labeled fMet-Phe-Phe-tRNA^Phe^ in the P site and had UAA stop-codon programmed A site. The mRNA sequence used to synthesize the peptide was GGGAAUUCGGGCCCUUGUUAACAAUUAAGGAGGUAUUAA**AUGUUCUUCUAA**UGCAGAAAAAAAAAAAAAAAAAAAAA (ORF underlined and bold, SD underlined). Class-1 release factors (RFs), overexpressed in *E. coli*, had mainly unmethylated glutamine (Q) in the GGQ motif and the RF2 variant contained Ala in position 246. Rate constants for conformational changes of RFs in response to cognate A-site stop codon (k_conf_) and for ester bond hydrolysis (k_hydr_) at different OH^−^ concentrations were estimated as described^22^. In short, purified release complexes (0.02 µM final concentration) were reacted at 25 °C with saturating amounts of RFs (0.8 µM final) in a quench-flow instrument, and the reaction stopped at different time points by quenching with 17% (final concentration) formic acid. Precipitated [^3^H]fMet-Phe-Phe-tRNA^Phe^ was separated from the soluble [^3^H]fMet-Phe-Phe peptide by centrifugation. The amounts of tRNA-bound and free peptides were quantified by scintillation counting of the ^3^H radiation. Reaction buffer was polymix-HEPES with free Mg^2+^ concentration adjusted from 5 to 2.5 mM by addition of 2.5 mM Mg^2+^-chelating UTP. The rate constants for RF association to the A site at 25 °C, k_a25_, were estimated from their previously published values at 37 °C, k_a37_ = 60 µM^−1^ s^−1^ for RF1 and 23 µM^−1^ s^−1^ for RF2^38^ through k_a25_ = (T_25_/ŋ_25_)·(ŋ_37_/T_37_), where T is the absolute temperature and ŋ the water viscosity. Kinetics simulations were carried out with the termination reaction steps modeled as consecutive first-order reactions^43^.

### Preparation of EM grids and time-resolved cryo-EM

Quantifoil R1.2/1.3 grids with a 300 mesh size were subjected to glow discharge in H_2_ and O_2_ for 25 s using a Solarus 950 plasma cleaning system (Gatan, Pleasanton, CA) set to a power of 10 W. Release complexes and RFs were prepared in the same way as for quench-flow experiments, except the release complexes were unlabeled. For each time point (24 ms and 60 ms), 4 µM of release complexes in polymix-HEPES with 2.5 mM UTP and 6 µM of class-1 release factors in the same buffer were injected into the corresponding microfluidic chip at a rate of 3 µl/s such that they could be mixed and sprayed onto a glow-discharged grid as previously described^33^. The final concentration of the release complexes and the class-1 release factors after rapid mixing in our microfluidic chip was 2 µM and 3 µM, respectively. As the mixture was sprayed onto the grid, the grid was plunge-frozen in liquid ethane-propane mixture (37%:63%) and stored in liquid nitrogen until it was ready to be imaged.

### Preparation of EM grids and blotting-plunging cryo-EM

Grids of RC_0_ and long-incubation complex were prepared with the following protocol. 3 uL sample was applied in the holey grids (gold grids R0.6/1 300 mesh, which was plasma cleaned using the Solarus 950 advanced plasma cleaning system (Gatan, Pleasanton, CA) for 25 s at 10 W using hydrogen and oxygen plasma). Vitrification of samples was performed in a Vitrobot Mark IV (FEI company) at 4 °C and 100% relative humidity by blotting the grids once for 6 s with a blot force 3 before plunging them into the liquid ethane-propane mixture.

### Cryo-EM data collection

Time-resolved cryo-EM grids were imaged either with a 300 kV Tecnai Polara F30 TEM or a Titan Krios TEM. The images were recorded at a defocus range of −1 to −3 µm on a K2 direct detector camera (Gatan, Pleasanton, CA) operating in counting mode with pixel size at 1.66 Å or 1.05 Å. A total of 40 frames were collected with an electron dose of 8 e^−^/pixel/s for each image. Blotting-plunging cryo-EM grids were imaged with a 300 kV Tecnai Polara F30 TEM. The images were recorded at a defocus range of 1–3 µm on a K2 direct detector camera (Gatan, Pleasanton, CA) operating in counting mode with pixel size at 1.24 Å. A total of 40 frames were collected with an electron dose of 8 e^−^/pixel/s for each image.

### Cryo-EM data processing

The beam-induced motion of the sample and the instability of the stage due to thermal drift was corrected using the MotionCor2 software program^44^. The contrast transfer function (CTF) of each micrograph was estimated using the CTFFIND4 software program^45^. Imaged particles were picked using the Autopicker algorithm included in the RELION 2.0 software program^46^. For each time point (Supplementary Figs 6 and  7), 2D classification of the recorded images were used to separate 70S ribosome-like particles from ice-like and/or debris-like particles picked by the Autopicker algorithm and to classify the particles that were picked for further analysis into 70S ribosome-like particle classes. These particle classes were then combined into a single dataset of 70S ribosome-like particles and subjected to a round of 3D classification for the purpose of eliminating those obvious contaminants from the rest of the dataset. This classification was set for 10 classes with the following sampling parameters: Angular sampling interval of 15°, offset search range of 5 pixels and offset search step of 1 pixel. The sampling parameters were progressively narrowed in the course of the 50 classification iterations, down to 3.7° for the angular sampling interval. At the end of the first classification round, two classes were found inconsistent with the known structure of the 70S ribosomes and were thus rejected. The rest of the particles were regrouped together as one class. All particles from this class were re-extracted using unbinned images. A consensus refinement was calculated using these particles. The A site of the 70S ribosome displays fractioned density indicating heterogeneity, then, therefore, the signal subtraction approach was applied. The A-site density was segmented out of the ribosome using Segger in Chimera^47^. The mass of density identified as release factor was used for creating a mask in RELION with 3 pixels extension and 6 pixels soft edge using relion_mask_create. This mask was used for subtracting the release factor-like signal from the experimental particles. The new particles images were used directly as input in the masked classification run with the number of particles set for ten classes, and with the mask around the release factor-binding region. This run of focused classification resulted in two separate classes, one with compact and one with extended conformation of the release factors. The corresponding raw particles were finally used to calculated consensus refinements. The local resolution of the final maps was computed using ResMap^48^.

For the RC_0_ complex dataset, the software MotionCor2^44^ was used for motion correction and dose weighting. Gctf^49^ was used for estimation of the contrast transfer function parameters of each micrograph. RELION^46^ was used for all other image processing steps. Particles picking was done automatically in RELION. Boxed out particles were extracted from dose-weighted micrographs with eight times binning. 2D classifications were initially performed on bin8 particle stacks to remove false positive particles from the particle picking step. 3D classification were performed on bin4 particle stacks. Classes from bin4 and bin2 3D classification showing high-resolution features were saved for further processing steps. Un-binned particles from this class were re-extracted and subjected to auto-refinement. The final density map was sharpened by applying a negative B-factor estimated by automated procedures. Local resolution variations were estimated using ResMap^48^ and visualized with UCSF Chimera^47^.

### Model building and refinement

Models of the *E. coli* 70S ribosome (5MDV, 5MDW, and 5DFE) were docked into the maps using UCSF Chimera. The pixel size was calibrated by creating the density map from the atomic model and changing the pixel size of the map to maximize the cross-correlation value. For the compact RF1 model, a homology model was generated with the crystal structure of the RF1 (PDB ID: 1ZBT) as a template using the SWISS-MODEL online server^50^. This homology model was rigid-body-fitted into the map using UCSF Chimera, followed by manual adjustment in Coot^51^. Due to the lack of density, domain I of RF1 was not modeled.

### Figure preparation

All figures showing electron densities and atomic models were generated using UCSF Chimera^47^.

## Supplementary information

Supplementary information

Peer Review

Reporting summary

Source Data

## Data Availability

The data that support the findings of this study are available from the corresponding author upon request. The atomic coordinates and the associated maps have been deposited in the PDB and EMDB with the accession codes 20173, 20184, 20187, 20188, 20193, 20204, 6ORE, 6ORL, 6OSQ, 6OST, 6OT3, and 6OUO. The source data underlying Supplementary Figs 2 and 3 are provided as a Source Data file.
