# Supplementary material for: The structural basis for release-factor activation during translation termination revealed by time-resolved cryogenic electron microscopy
Source: Nat Commun. 2019 Jun 12;10:2579. doi: 10.1038/s41467-019-10608-z (PMC6561943; doi:10.1038/s41467-019-10608-z)
Supplement: Supplementary file 1 — Supplementary information [file 41467_2019_10608_MOESM1_ESM.pdf]

Supplementary Information

The structural basis for release factor activation during translation termination revealed by time-resolved cryogenic electron microscopy

Fu et al.

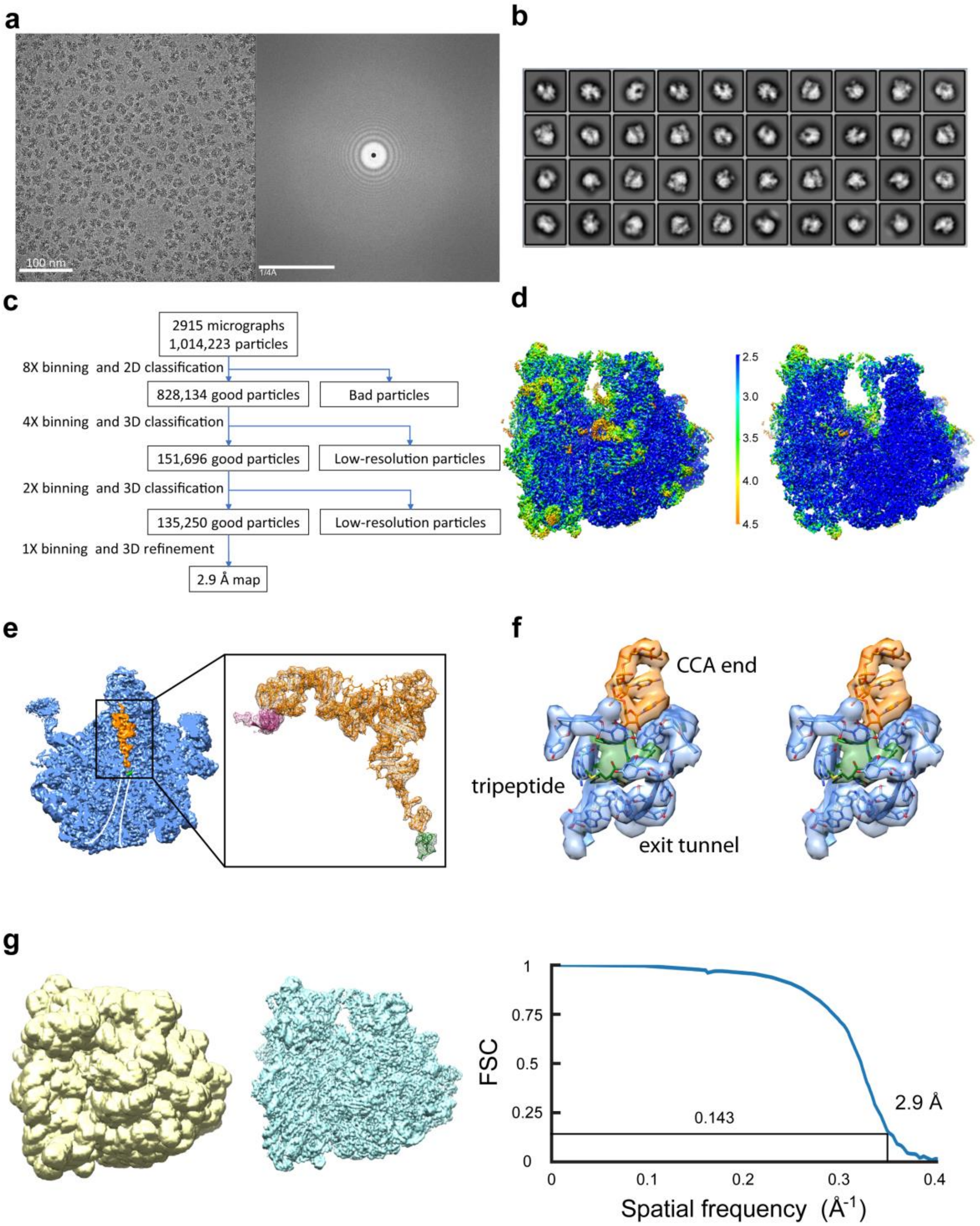

Supplementary Figure 1. Cryo-EM data processing of release complex, RC$_0$. a. A representative micrograph collected with a Polara transmission electron microscope with the corresponding power spectrum. **b.** Representative 2D class averages from reference-free 2D classification. **c.** Particle classification and structural refinement procedures used. **d.** Local resolution estimation of the cryo-EM density map. The density map of release complex is displayed in surface representation, and colored according to the local resolution (see color bar). **e.** The ribosomal exit tunnel (white line) in 50S

ribosomal subunit (blue) is occupied by programed tripeptide (green) attached to P-site tRNA (orange). The interaction between mRNA (pink) and tRNA is shown in zoom-in view on the right. **f.** Wall-eye stereoview of part of the CCA end, tripeptide, and ribosome exit tunnel. **g.** The mask used for resolution estimation (left) including all components of the reconstruction (middle). FSC curve for cryo-EM reconstruction (right).

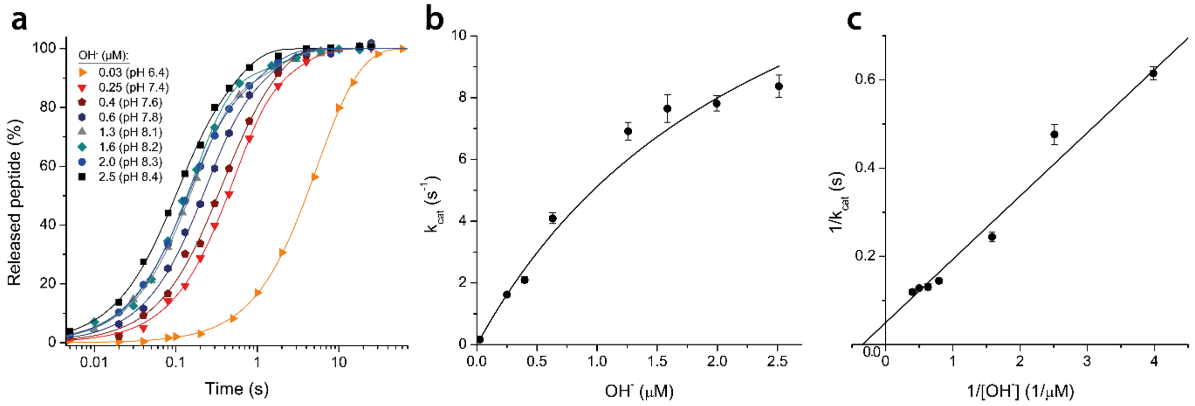

Supplementary Figure 2. The maximal rate (k_{cat}) of peptide release by RF1 has a pH-independent and a pH-dependent step. a. Extent of RF1-dependent fMet-Phe-Phe peptide release from P-site bound fMet-Phe-Phe-tRNA (y axis) versus time (x axis) at different hydroxyl ion (OH^-) concentrations. The experiments were performed at 25°C with 0.02 μM release complexes and saturating (0.8 μM) RF1 concentration (k_{cat}-range) (Methods). **b.** Maximal rate (k_{cat}) of peptide release (y-axis) increased to a plateau value (k_{conf} = 18.1 ± 3.3 s^{-1}) with increasing [OH^-] (x-axis), which led to the hypothesis that the maximal rate of peptide release is limited by a pH-independent change of RF conformation at high pH[8]. Error bars represent s.d. from three replicates. The Michaelis-Menten model, used to obtain k_{conf}, was compared to a linear model (k_{cat} linearly increasing with OH^- concentration) using the F-test. The linear model could be rejected ($F_{5,6}$ = 35, P < .001). **c.** Lineweaver-Burk plot of 1/k_{cat} versus 1/[OH^-] from the data in Supplementary Figure 2b. The value at 1/0.025 μM OH^- (x = 40; y = 6) is not shown for better visibility of the remaining data points. The plateau value k_{conf} was obtained from the plot including all values as 1/y-intercept and was 16.7 ± 3.6 s^{-1}. Source data are provided as a Source Data file.

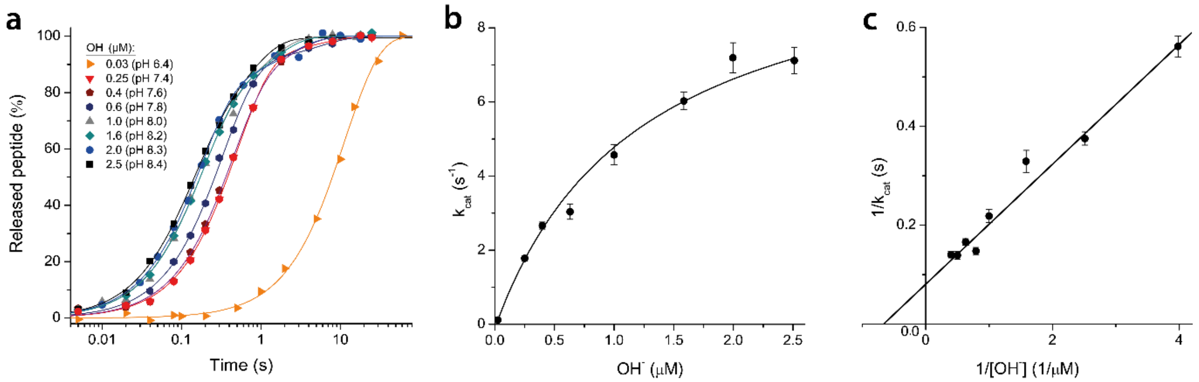

Supplementary Figure 3. The maximal rate (k_{cat}) of peptide release by RF2 has a pH-independent and a pH-dependent step. a. Extent of RF2-dependent fMet-Phe-Phe peptide release from P-site bound fMet-Phe-Phe-tRNA (y axis) versus time (x axis) at different hydroxyl ion (OH^-) concentrations. The experiments were performed at 25°C with 0.02 µM release complexes and saturating (0.8 µM) RF2 concentration (Methods). **b.** Maximal rate (k_{cat}) of peptide release (y-axis) by RF2 increased to a plateau value (k_{conf} = 10.8 ± 0.9 s^{-1}) with increasing [OH^-] (x-axis). Error bars represent s.d. from three or four replicates. The Michaelis-Menten model, used to obtain k_{conf}, was compared to a linear model (k_{cat} linearly increasing with OH^- concentration) using the F-test. The linear model could be rejected ($F_{5,6}$ = 141, $P < .001$). **c.** Lineweaver-Burk plot of 1/k_{cat} versus 1/[OH^-] from the data in Supplementary Figure 3b. The value at 1/0.025 µM OH^- (x = 40; y = 8.5) is not shown for better visibility of the remaining data points. The plateau value k_{conf} was obtained from the plot including all values as 1/y-intercept and was 12.5 ± 1.2 s^{-1}. Source data are provided as a Source Data file.

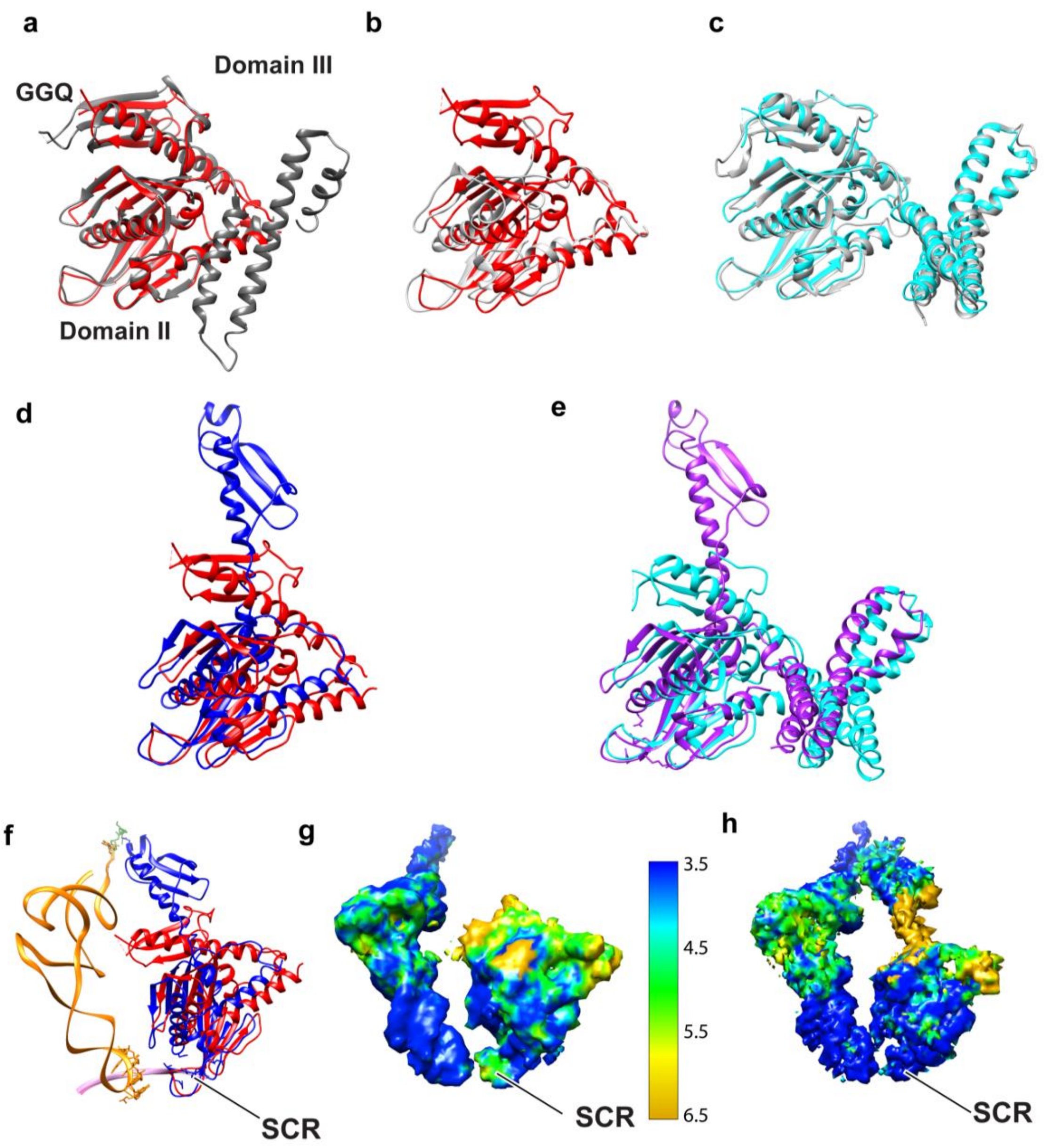

Supplementary Figure 4. Comparison of RF. a. Comparison of compact RF1 from pre-accommodated ribosome complex (red) and free RF1 in the crystal form (dim grey, 1RQ0). **b.** Comparison between RF1 in compact state from 24 ms (red) and BlaS halted RF1 (grey, 6BOK). **c.** Overlay between RF2 in compact state from 24 ms (cyan) and ArfA-RF2 compact state (light grey, 5U9G). **d.** Comparison between RF1 in compact state from 24 ms (red) and RF1 in extended state from 60 ms (blue), aligned using 16S r . **e.** Comparison between RF2 in compact state from 24 ms (cyan) and RF2 in extended state from 60 ms (purple). **f.** Comparison of RF1 in compact state (red) and RF1 in extended state (blue) from 24 ms and 60 ms, respectively. tRNA (orange), mRNA (pink) and peptide (green) are shown. **g and h.** Local resolution estimation for tRNA, peptide and RF1 by Resmap. The resolution of SCR in compact state is lower than that in open state, indicating loose binding. The alignments are done with 16S rRNA as reference.

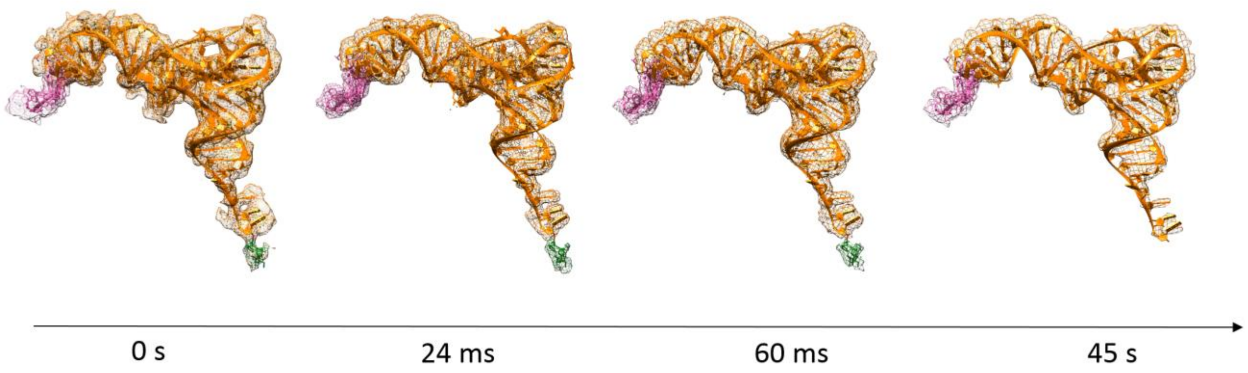

Supplementary Figure 5. Tripeptide hydrolysis and release. mRNA, P-tRNA and tripeptide density with refined models of RC_0 from 0 ms, compact RF1 bound pre-accommodated ribosome from 24 ms, extended RF1-bound accommodated ribosome from 60 ms, and extended RF1-bound accommodated ribosome from 45 s, respectively (left to right). Pink: mRNA; orange: tRNA; and green: tripeptide.

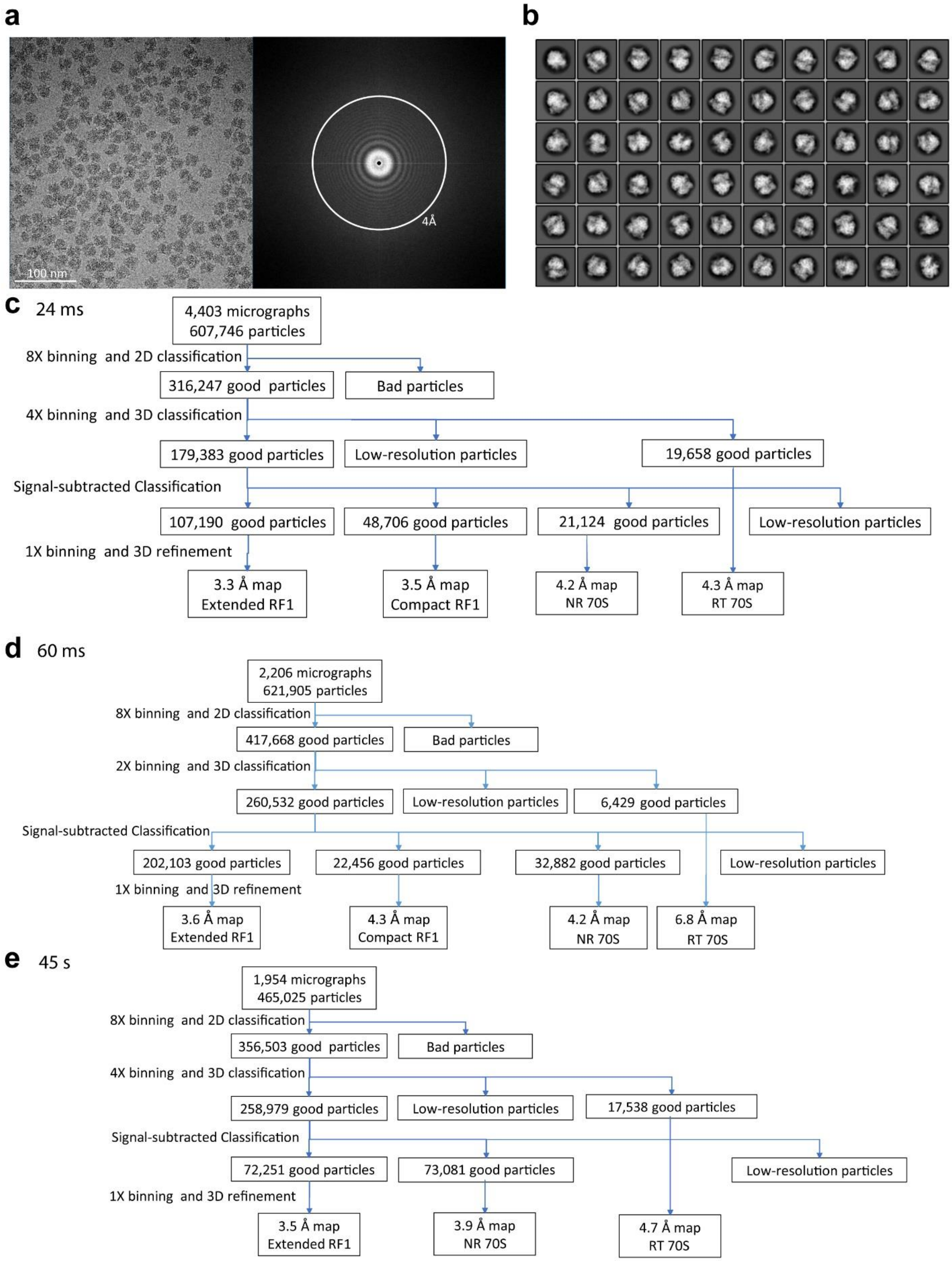
a
100 nm
4Å
b
c
24 ms
4,403 micrographs
607,746 particles
8X binning and 2D classification
316,247 good particles
Bad particles
4X binning and 3D classification
179,383 good particles
Low-resolution particles
19,658 good particles
Signal-subtracted Classification
107,190 good particles
48,706 good particles
21,124 good particles
Low-resolution particles
1X binning and 3D refinement
3.3 Å map
Extended RF1
3.5 Å map
Compact RF1
4.2 Å map
NR 70S
4.3 Å map
RT 70S
d
60 ms
2,206 micrographs
621,905 particles
8X binning and 2D classification
417,668 good particles
Bad particles
2X binning and 3D classification
260,532 good particles
Low-resolution particles
6,429 good particles
Signal-subtracted Classification
202,103 good particles
22,456 good particles
32,882 good particles
Low-resolution particles
1X binning and 3D refinement
3.6 Å map
Extended RF1
4.3 Å map
Compact RF1
4.2 Å map
NR 70S
6.8 Å map
RT 70S
e
45 s
1,954 micrographs
465,025 particles
8X binning and 2D classification
356,503 good particles
Bad particles
4X binning and 3D classification
258,979 good particles
Low-resolution particles
17,538 good particles
Signal-subtracted Classification
72,251 good particles
73,081 good particles
Low-resolution particles
1X binning and 3D refinement
3.5 Å map
Extended RF1
3.9 Å map
NR 70S
4.7 Å map
RT 70S

f

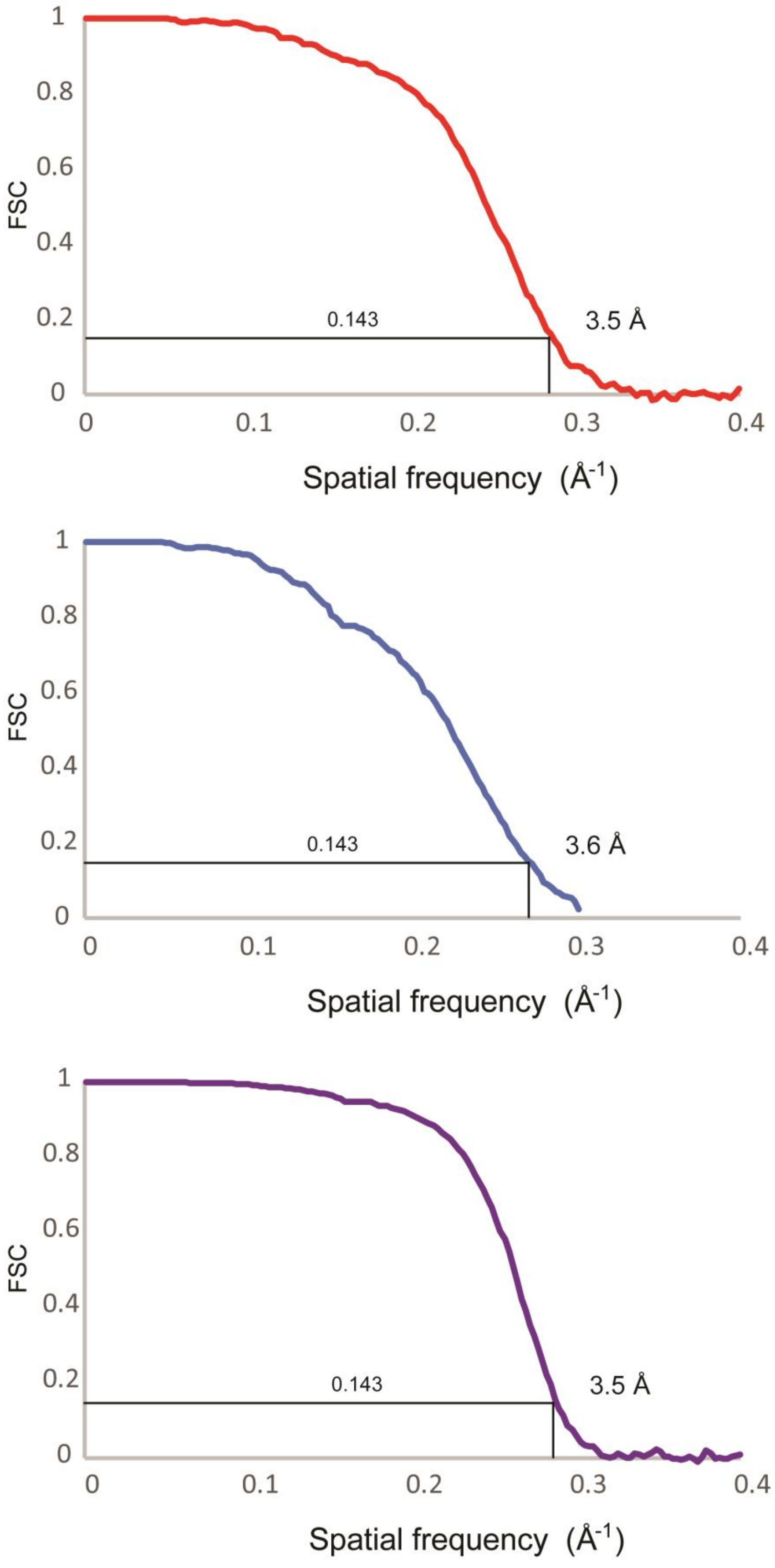

FSC
1
0.8
0.6
0.4
0.2
0
0.143
3.5 Å
0
0.1
0.2
0.3
0.4
Spatial frequency (Å^{-1})
FSC
1
0.8
0.6
0.4
0.2
0
0.143
3.6 Å
0
0.1
0.2
0.3
0.4
Spatial frequency (Å^{-1})
FSC
1
0.8
0.6
0.4
0.2
0
0.143
3.5 Å
0
0.1
0.2
0.3
0.4
Spatial frequency (Å^{-1})

Supplementary Figure 6. Cryo-EM data processing of RF1-bound ribosome ensembles at 24 ms, 60 ms, and 45s. a. A representative micrograph with corresponding power spectrum. **b.** Representative 2D class averages from reference-free 2D classification. **c, d, and e.** Particle classification and structural refinement procedures used for 24 ms (c), 60 ms (d), and 45 s (e) data sets. **f.** FSC curves for cryo-EM reconstructions of compact RF1 bound pre-accommodated ribosome from 24 ms (red), extended RF1 bound accommodated ribosome from 60 ms (blue), and extended RF1 bound accommodated ribosome from 45 s (purple), respectively.

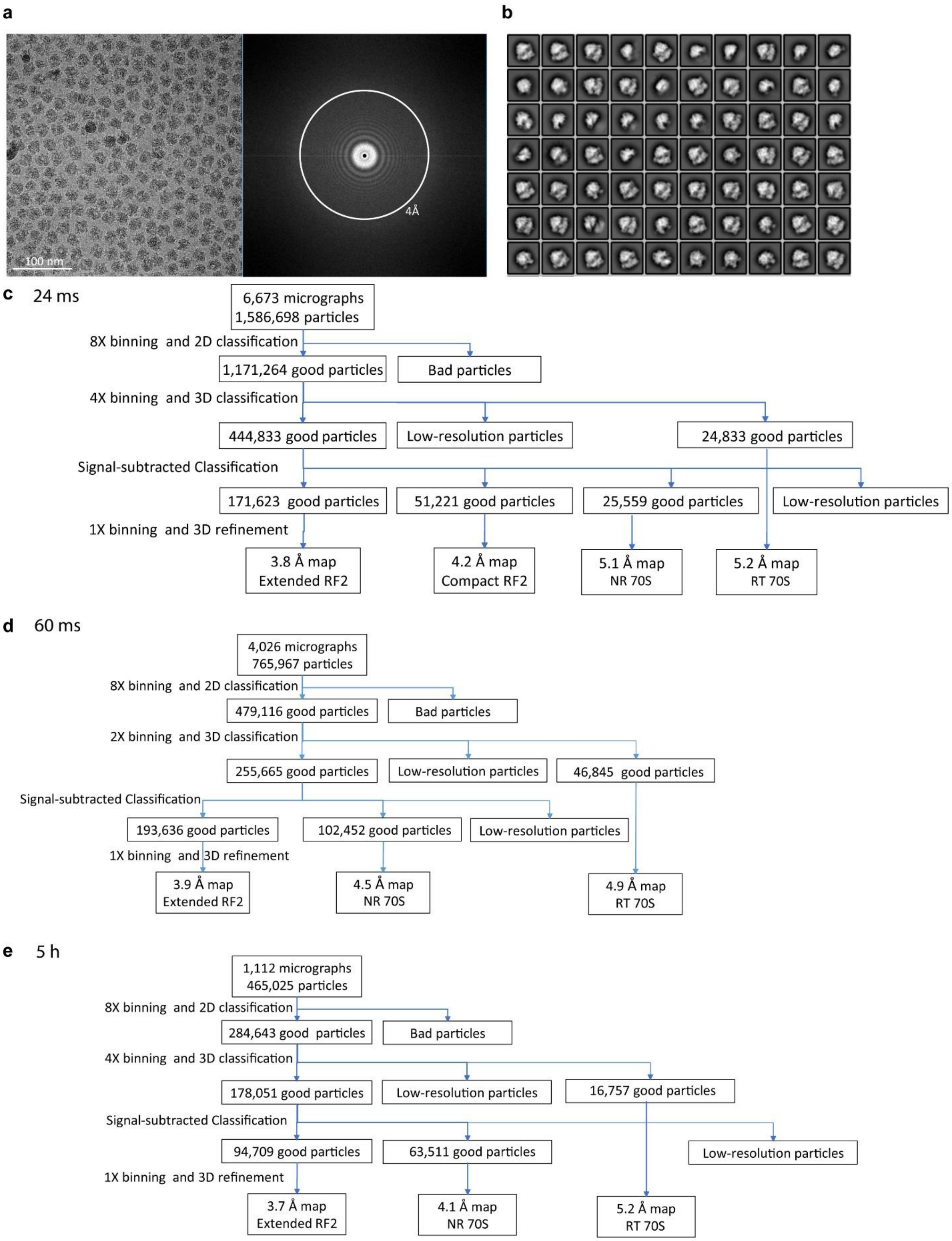

a
100 nm
4Å
b
c 24 ms
6,673 micrographs
1,586,698 particles
8X binning and 2D classification
1,171,264 good particles
Bad particles
4X binning and 3D classification
444,833 good particles
Low-resolution particles
24,833 good particles
Signal-subtracted Classification
171,623 good particles
51,221 good particles
25,559 good particles
Low-resolution particles
1X binning and 3D refinement
3.8 Å map Extended RF2
4.2 Å map Compact RF2
5.1 Å map NR 70S
5.2 Å map RT 70S
d 60 ms
4,026 micrographs
765,967 particles
8X binning and 2D classification
479,116 good particles
Bad particles
2X binning and 3D classification
255,665 good particles
Low-resolution particles
46,845 good particles
Signal-subtracted Classification
193,636 good particles
102,452 good particles
Low-resolution particles
1X binning and 3D refinement
3.9 Å map Extended RF2
4.5 Å map NR 70S
4.9 Å map RT 70S
e 5 h
1,112 micrographs
465,025 particles
8X binning and 2D classification
284,643 good particles
Bad particles
4X binning and 3D classification
178,051 good particles
Low-resolution particles
16,757 good particles
Signal-subtracted Classification
94,709 good particles
63,511 good particles
Low-resolution particles
1X binning and 3D refinement
3.7 Å map Extended RF2
4.1 Å map NR 70S
5.2 Å map RT 70S

f

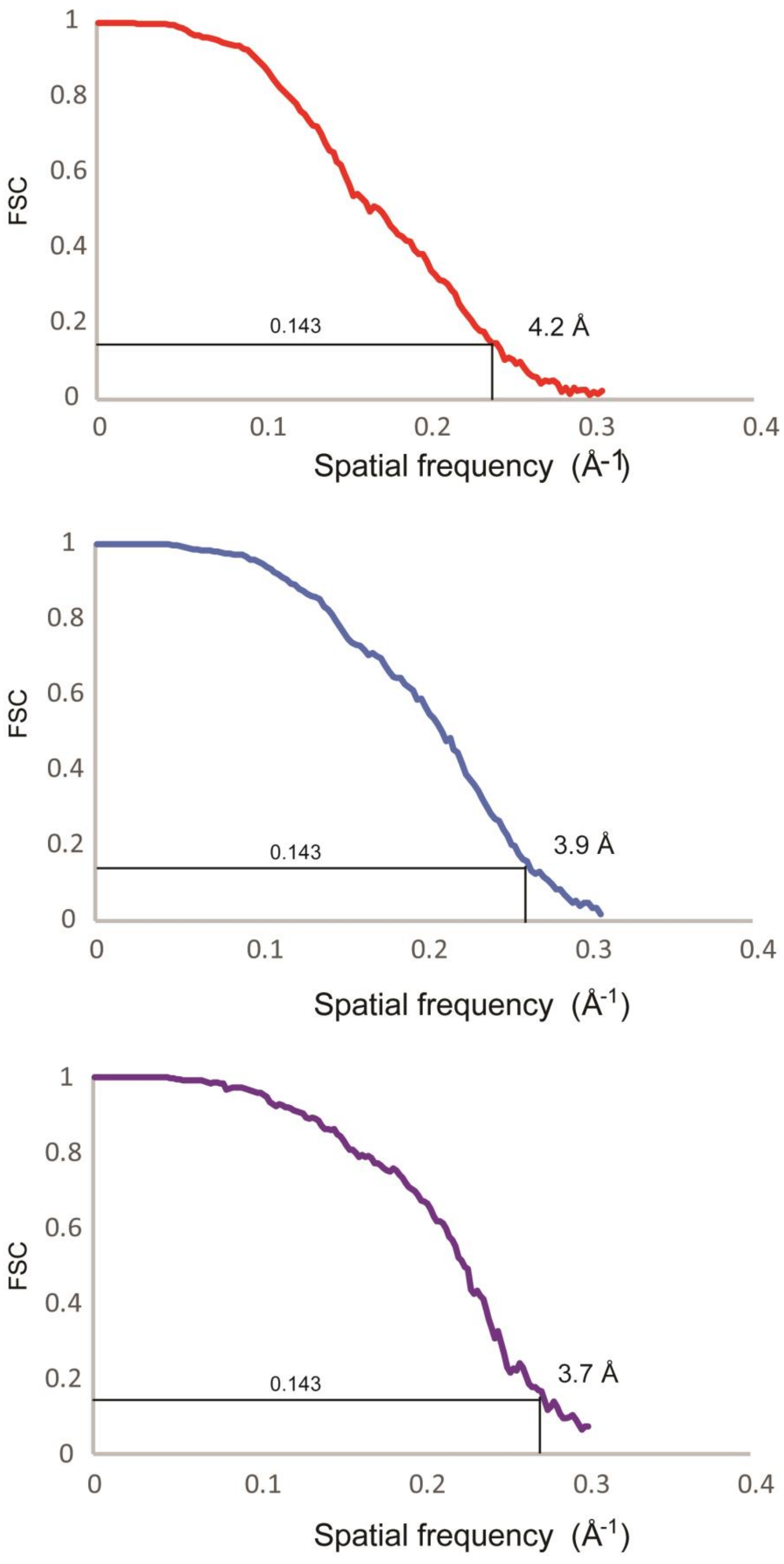

1
0.8
0.6
0.4
0.2
0
FSC
0.143
4.2 Å
0
0.1
0.2
0.3
0.4
Spatial frequency (Å-1)
1
0.8
0.6
0.4
0.2
0
FSC
0.143
3.9 Å
0
0.1
0.2
0.3
0.4
Spatial frequency (Å-1)
1
0.8
0.6
0.4
0.2
0
FSC
0.143
3.7 Å
0
0.1
0.2
0.3
0.4
Spatial frequency (Å-1)

Supplementary Figure 7. Cryo-EM data processing of RF2-bound ribosome ensembles at 24 ms, 60 ms, and 5h. a. A representative micrograph with corresponding power spectrum. **b.** Representative 2D class averages from reference-free 2D classification. **c,d, and e.** Particle classification and structural refinement procedures used for 24 ms (c), 60 ms (d), and 5 h (e) data sets. **f.** FSC curves for cryo-EM reconstructions of compact RF2 bound pre-accommodated ribosome from 24 ms (red), extended RF2 bound accommodated ribosome from 60 ms (blue), and extended RF2 bound accommodated ribosome from 5 h (purple), respectively.

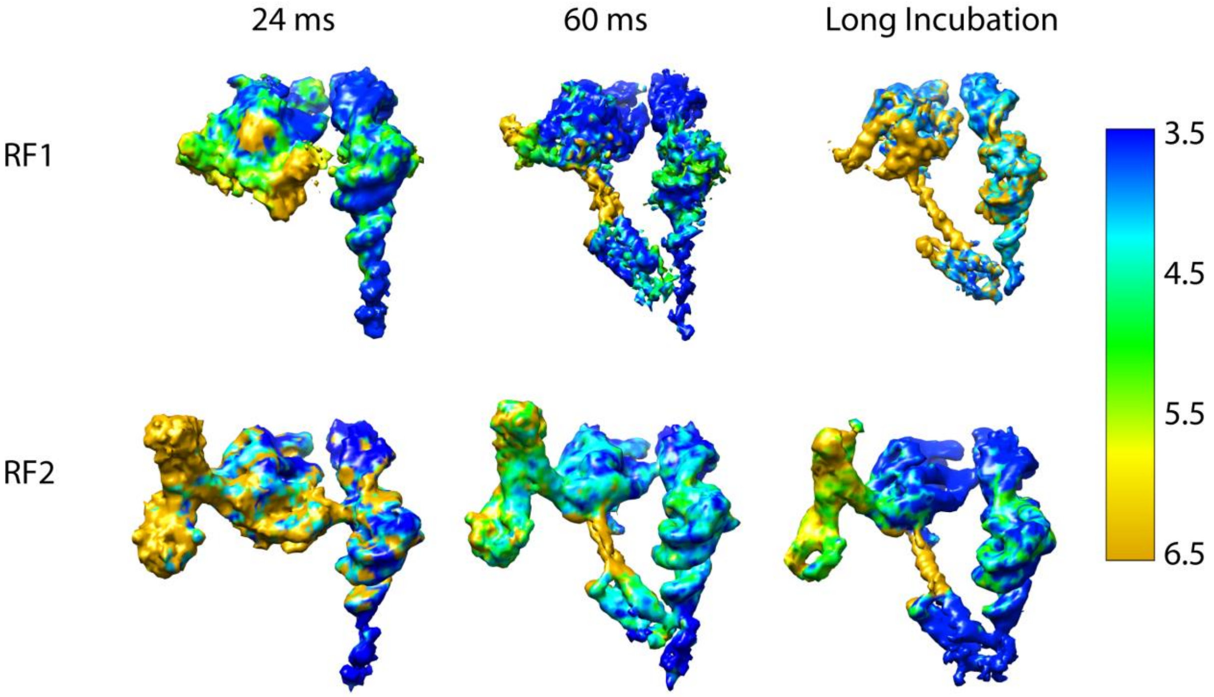

Supplementary Figure 8 Local resolution of RFs. RF1, RF2, tRNA and peptide are colored based on their local resolution estimated by Resmap. The scale bar is shown on the right and the unit is A.

Supplementary Table 1. Cryo-EM data collection and model statistics of ribosome complexes.

	release complex	pre-accommodated	accommodated	accommodated	pre-accommodated	accommodated	accommodated
**Data Collection**							
Electron microscope	Polora	Krios	Polora	Polora	Polora	Polora	Polora
Pixel size (Å)	1.24	1.04	1.66	1.66	1.66	1.66	1.66
Particle number	135250	48706	202103	72251	51221	193636	94709
Time point	0s	24 ms	60 ms	45 s	24 ms	60 ms	5h
Release factor	unbound	RF1	RF1	RF1	RF2	RF2	RF2
Map resolution (Å)	2.9	3.5	3.6	3.5	4.2	3.9	3.7
**Refinement**							
Model-to-map	0.9002	0.8697	0.8485	0.8695	0.8762	0.8719	0.8471
**R.M.S Deviation**							
Bond lengths (Å)	0.008	0.009	0.008	0.008	0.009	0.006	0.008
Bond angles	0.758	0.881	0.843	0.877	0.999	0.822	0.937
**Ramachandran plot**							
Outliers	0.04%	0.03%	0.05%	0.03%	0.03%	0.03%	0.03%
Allowed	3.55%	6.09%	5.27%	5.56%	6.86%	5.08%	6.19%
Favored	96.41%	93.88%	94.68%	94.41%	93.11%	94.88%	93.78%
Rotamer outliers	0.39%	0.19%	0.37%	0.41%	0.18%	0.18%	0.49%
Cbeta deviations	0%	0%	0%	0%	0%	0%	0%
