# Supplementary material for: The structural basis for release-factor activation during translation termination revealed by time-resolved cryogenic electron microscopy
Source: Nat Commun. 2019 Jun 12;10:2579. doi: 10.1038/s41467-019-10608-z (PMC6561943; doi:10.1038/s41467-019-10608-z)
Supplement: Supplementary file 2 — Peer Review [file 41467_2019_10608_MOESM2_ESM.pdf]

Reviewers' comments:

Reviewer #1 (Remarks to the Author):

During translation termination in bacteria, RF1 or RF2 recognize the stop codon in the mRNA and catalyze release of the nascent polypeptide chain from the P-site tRNA. While there are many structures of termination complexes with RFs in an accommodated state, the pre-accommodated compact conformation of RFs has only been observed in the context of ribosome rescue of truncated mRNAs by ArfA and RF2, as well as more recently during canonical termination but employing a hyper-accurate RF1 variant.

Here the authors use time-resolved cryo-EM to visualize the conformation of RF1 and RF2 in pre-accommodated and accommodated states. At 20ms, a compact conformation of RFs is visualized on the ribosome that converts to an accommodated state with the GGQ motif at the PTC at 60ms. Surprisingly, at 60ms the authors claim to still observe the nascent chain despite breakage of the ester linkage and only at later time-points (200ms) does the nascent chain dissociate from the ribosome.

The visualization of the compact pre-accommodated and subsequent accommodated states of RFs during a canonical termination reaction in a time-resolved manner is fantastic and provide important insights into a fundamental process, therefore I am very positive in the general interest of this manuscript to the readers of Nat Comm.

However, there are a large number of major points that need to be addressed before publication. The authors make many claims regarding the resolution but these cannot be substantiated based on the presented extended data. In fact, I am surprised about the low quality of many of the technical aspects of the paper.

1. For example, the correlation between the reported resolutions and the density images is poor...are the authors filtering the maps to lower resolutions, if so then this should be mentioned in the legend with the relevant details of how and to which resolution.

2. The FSC curve does not even go to zero in Extended Figure 1!! In fact, it appears to plateau at 0.25! One wonders how the resolution is determined at 0.143? Or are the authors using the O.5 criteria now like they did in the past? Either way the curve is not acceptable and the method of resolution calculation needs to be made clear

3. I am suspicious about the average resolution of 2.5A fort he RC0 complex since this is basically at Nyquist when the pixel size was 1.24A.

4. Extended Figure 6...again the FSC curve does not reach zero and the X-axis is truncated.

5. Extended Figure 7...The FSC curves simply look sick. One wonders if such graphs even allow gold standard criterion? But maybe the authors are not using it?

6. Regardless of the average resolution, the local resolution of the ligands in each of the complexes is the important factor. This is simply ignored, which is not acceptable. Images need to be provided at least for RF1 and RF2 with local resolution in the different complexes at the different time points. This is especially important for the reader to assess whether the authors really have the resolution to distinguish between a broken ester bond or not.

7. There is no discussion regarding the provocative data from the Rodnina group (Adio eLife 2018), where they observe rotated states for RF1 and especially RF2 with deacylated tRNA. This does not appear to be substantiated by the data here and should therefore be discussed? However, when

looking at the number of particles picked and ending up in the final reconstructions, one notices that in any cases up to 50% are discarded? One wonders if the authors are overlooking many states e.g. similar to Korostelev states or other rotated state? Its hard to believe that all these discarded particles are junk?

8. Likewise, the Rodnina group (Graf et al Nat Comm 2018) reported that L7/L12 interact with domain I to facilitate binding of RFs to the ribosome during termination. Surprisingly, this data is not even discussed despite being directly relevant to the story. Overall one gets the impression that the overall text is short and incomplete i.e. a Nature letter format and would benefit from being expanded to give credit to all the work related to the topic, rather than simply neglecting to even cite such works.

Reviewer #2 (Remarks to the Author):

The work presented in this paper is a great demonstration of the power of time-resolved Cryo-EM in structurally visualizing short-lived intermediates of biological processes. Here, it is used to resolve two conformational intermediates in the first 60ms of bacterial translation termination. The two intermediates were identified in structures of the 70S termination complex containing a P-site tripeptidyl tRNA, mRNA with UAA stop codon in the A site, and either RF1 or RF2 bound to the A site. The first intermediate (pre-accommodation state) shows the RF1/RF2 on the ribosome in the compact conformation similar to what is observed in solution ribosome-free or when bound to HemK methyltransferase. The second intermediate (accommodation state) shows the extended conformation of RF1/RF2 seen in previous crystal and Cryo-EM structures of termination complexes. This paper reports the first structural evidence of this conformational switch in the native pathway of translation termination. The structural basis of stop codon recognition and peptide release in this work contributes greatly to our understanding of the high decoding accuracy in termination and that makes this work compelling for publication to the broader audience of Nature Communications. But to do justice to the work presented, there are a several places in this paper that require further clarification/elaboration/correction that would add strength to this story.

The larger changes that I request are below:
1. In page 2 line 46 it is written “Stop codon reading by RFs is aided by class-2 RFs…” Please clarify the differences in the roles of the bacterial (RF3) and eukaryotic/archaea (eRF3/aRF3) class-2 RFs.
2. It is stated in page 3 line 112 that the Cryo-EM data show “somewhat faster conformational transition than in the quench-flow experiments”. Please offer an explanation for why could be the case.
3. Are there any differences in the densities between the 24ms and 60ms datasets for the 70S-RF1(extended) state?
4. After the phrase in page 4 line 128 “At a much later time-point” add in parentheses which specific dataset you are referring to (e.g. 45s data).
5. Explain what was observed in the structure to conclude that the SCR motif is “bound loosely” (page 5 line 176), such as poor local resolution of these residues, etc.
6. Accompany the section of the paper talking about the recently published Bl-S-halted RF1 structure (lines 171-193) with a figure comparing the compact RF1 structure from this study with that from the Bl-S-halted complex.
7. For Fig. 4, add reference to where the 200ms timepoint came from for the “peptide release RF-ribosome complex”

The smaller typographical changes are listed below:
1. Change “eRF1 and aRF1 factors read all –codons” to “eRF1 and aRF1 factors read all three stop codons” in page 2 line 46
2. Change “Gly-Gly-Glu triplet” to “Gly-Gly-Gln triplet” in page 2 line 50
3. Insert “of the bacterial 70S ribosome” after “70Å separating DC and PTC” in page 2 line 53

4. Add “Extended Data Fig. 5” to the figure reference “(Fig. 2e and 2f)” in page 4 line 128
5. Correct the “flipped-out nucleotides A1492 and A1913” in page 4 line 152 to “A1493 and A1913”
6. Add “Fig. 3b” to the figure reference “(Fig. 3d)” in page 4 line 152
7. Report the ratio of ethane-propane mixture in page 7 line 248
8. Replace “alkane” in page 8 line 256 with “ethane-propane mixture”
9. Correct the label in Fig. 3a and 3b from A530 to G530
10. Correct the x-axis of Extended Data Fig. 2b from “OH- (μM)” to “[OH-] (μM)”
11. Correct the Fig. 3d caption with “Gold: A1492 and A1493; and orange: S12”

Reviewer #3 (Remarks to the Author):

Fu and co-authors describe their biochemical and cryo-EM work on 70S translation termination by RF1 and RF2. An unresolved question in the field is how RF1 and RF2 bind the ribosome transiently to probe an mRNA codon in the A site. The field in general agrees that RFs bind to the ribosome in a compact conformation and undergo a large-scale conformational change if a stop codon is recognized. But compact states have not been captured on the ribosome except in truncated mRNA-70S-ArfA-RF2 complexes and in a hyperaccurate RF1-70S complex in the presence of blasticidin S.

In this work, quench-flow experiments suggested a time course for the conformational change in a two-step reaction of peptide release. Time-resolved cryo-EM captured compact RF1 and RF2 at near-atomic resolutions of higher than 4 A. These structures provide insights into the early stages of codon sampling by RF1 and RF2, which involve stop-codon preorganization by RF1/RF2 for docking of the switch loop and subsequent insertion of the catalytic GGQ motif into the peptidyl transferase center. In summary, this manuscript describes important intermediates of translation termination.

I strongly recommend this manuscript for publication. I suggest the authors address the following minor points:
1. Page 2, line #50. Correct “Gly-Gly-Glu” to “Gly-Gly-Gln”.
2. #62, a suggestion: change “GGQ-induction of ester bond hydrolysis” to “GGQ-mediated ester bond hydrolysis”
3. #66-67, on SAXS. The authors mention their early SAXS work, in which they interpreted free RF1 as “extended in bulk solution”. However other studies on RF1 and RF2 must be mentioned, which suggested a compact conformation of free RFs in solution:
a. SAXS (Zoldak et al; https://www.ncbi.nlm.nih.gov/pmc/articles/PMC1849895/)
b. FRET (Trappl and Joseph; https://www.ncbi.nlm.nih.gov/pubmed/26827724)
4. Page, 3. #79 and all other instances of “Bl-S”: replace “Bl-S” (which is confusing as it may be read as “B-one-S”) with “BlaS”. “Blasticidin S” is shortened to “BlaS” in numerous research papers, while “Bl-S” is not commonly used.
5. While RF2 is discussed in text, no figures are shown with RF2 structures. Also, it is hard to see how compact RF1 and RF2 are positioned on the ribosome relative to extended RF1 and RF2, respectively. To fill these gaps, add three (or more) figures/panels showing:
a. Alignment of compact and extended RF1 (aligned using 16S rRNA), in a view similar to figure panels 2c and 2d overlaid.
b. Alignment of compact and extended RF2 (aligned on 16S rRNA)
c. Alignment of compact conformations of RF2 in this work and in the ArfA-bound 70S complex (aligned on 16S rRNA).

Reviewer 1

During translation termination in bacteria, RF1 or RF2 recognize the stop codon in the mRNA and catalyze release of the nascent polypeptide chain from the P-site tRNA. While there are many structures of termination complexes with RFs in an accommodated state, the pre-accommodated compact conformation of RFs has only been observed in the context of ribosome rescue of truncated mRNAs by ArfA and RF2, as well as more recently during canonical termination but employing a hyper-accurate RF1 variant.

Here the authors use time-resolved cryo-EM to visualize the conformation of RF1 and RF2 in pre-accommodated and accommodated states. At 20ms, a compact conformation of RFs is visualized on the ribosome that converts to an accommodated state with the GGQ motif at the PTC at 60ms. Surprisingly, at 60ms the authors claim to still observe the nascent chain despite breakage of the ester linkage and only at later time-points (200ms) does the nascent chain dissociate from the ribosome.

The visualization of the compact pre-accommodated and subsequent accommodated states of RFs during a canonical termination reaction in a time-resolved manner is fantastic and provide important insights into a fundamental process, therefore I am very positive in the general interest of this manuscript to the readers of Nat Comm. However, there are a large number of major points that need to be addressed before publication. The authors make many claims regarding the resolution but these cannot be substantiated based on the presented extended data. In fact, I am surprised about the low quality of many of the technical aspects of the paper.

1. For example, the correlation between the reported resolutions and the density images is poor...are the authors filtering the maps to lower resolutions, if so then this should be mentioned in the legend with the relevant details of how and to which resolution.

We thank Reviewer 1 for these suggestions. In response, we added a figure of the CCA end, tripeptide and part of ribosome exit tunnel density in the extended figure 1f, and we replaced extended figure 1e with the unfiltered density map.

2. The FSC curve does not even go to zero in Extended Figure 1!! In fact, it appears to plateau at 0.25! One wonders how the resolution is determined at 0.143? Or are the authors using the O.5 criteria now like they did in the past? Either way the curve is not acceptable and the method of resolution calculation needs to be made clear.

We thank reviewer 1 for pointing this out. In response we now use a more generous mask that lets the FSC fall to zero, and the reported resolution of the Ro complex is now 2.9 A. We show the local resolution estimation using Resmap in the expanded data fig. 1.

3. I am suspicious about the average resolution of 2.5A for the RC_0 complex since this is basically at Nyquist when the pixel size was 1.24A.

With the use of the new mask, the reported resolution for the RC0 complex is now 2.9 A. We have now also updated the extended data table 1.

4. Extended Figure 6...again the FSC curve does not reach zero and the X-axis is truncated.

We have updated the FSC curve figure in extended data figure 6.

5. Extended Figure 7...The FSC curves simply look sick. One wonders if such graphs even allow gold standard criterion? But maybe the authors are not using it?

We thank the reviewer for pointing this out. We have updated the FSC curves in this figure, and note that the FSC curves shown in the previous version of the ms were not the correct ones.

6. Regardless of the average resolution, the local resolution of the ligands in each of the complexes is the important factor. This is simply ignored, which is not acceptable. Images need to be provided at least for RF1 and RF2 with local resolution in the different complexes at the different time points. This is especially important for the reader to assess whether the authors really have the resolution to distinguish between a broken ester bond or not.

In response to the comments of Reviewer 1 on the local resolution of factors, we have calculated the local resolution of each complex (RF1 and RF2 from 24 ms, 60 ms and the long incubation time point). This information is now available as extended data fig 8.

7. There is no discussion regarding the provocative data from the Rodnina group (Adio eLife 2018), where they observe rotated states for RF1 and especially RF2 with deacylated tRNA. This does not appear to be substantiated by the data here and should therefore be discussed?

Here is an account of the similarities and differences between our results and theirs in Adio eLife 2018:

First, in our study, the termination complex (in the control experiment) is indeed biochemically similar to the pre-hydrolysis complexes (PreHC) from the Adio eLife 2018 paper, except that we have three amino-acid attached to the tRNA while they only have one or two. This appears to be an important difference, as pointed out below.

Without RF1/RF2, we did not observe any rotated state of the ribosome, as shown in our control experiment, while they observed a small fraction of rotated-state ribosomes in their smFRET experiment. We believe the reason for this discrepancy is that we used the tripeptide rather than single peptide or dipeptide. The P/E position of tRNA may accommodate one or two amino acids but if there are three amino acids attached to the tRNA, the P/E position will result in a clash between the tripeptide and the 23S rRNA. In fact, all the studies the authors cited that report rotated-state ribosomes used acylated tRNA with one amino acid only ((Cornish et al., 2008; Fischer et al., 2010; Ling and Ermolenko, 2015)).

Second, prompted by the referee's question, we performed an additional careful classification, and found a class of rotated-state ribosomes in all 24ms, 60ms and long-incubation experiments. We now added the finding to the Extended Data Fig. 6 and 7. This rotated-state ribosome has no RF1/RF2 bound.

Finally, we did not include RF3 as part of the investigation because the stop codon recognition and the conformational change of RF1/2 were our focus. Therefore, it is not possible to compare their RF3-modulated rotated state with our results.

We added one paragraph before the Conclusion to discuss the relevance of the Adio et al. paper:

"Another recently published study using smFRET [41] reported two states of the termination complex, non-rotated and rotated, in apparent contradiction to our results as we only found one, non-rotated state. However, the discrepancy can be explained by the fact that the authors used a termination complex with the P-site tRNA bound with one or two aminoacids, while we used tRNA bound with three. The tripeptide bound to the tRNA prevents the intersubunit rotation as the P/E position associated with it would produce a strong steric clash with the 23S

rRNA. The mono- or di-peptide-bound tRNAs, in contrast, will allow formation of the P/E position to some degree, and thereby allow rotation in a fraction of the ribosomes. This explanation is also supported by the authors' own results as the observed fraction of ribosomes in the rotated state is dependent on the identity of mono-peptide and di-peptide, with most found for the mono-peptide."

However, when looking at the number of particles picked and ending up in the final reconstructions, one notices that in any cases up to 50% are discarded? One wonders if the authors are overlooking many states e.g. similar to Korostelev states or other rotated state?

We used focused classification and partial signal subtraction followed by classification to reprocess the factoring binding complexes. But we did not see any other states other than we reported.

Its hard to believe that all these discarded particles are junk?

In our data processing, we usually prefer picking false positives in excess so as not to miss but a very minor fraction of true particles. However, in the 2D classification, it is easy to remove virtually all false positives, which we here have referred to as bad particles, because they consist of ice blobs or carbon edges.

8. Likewise, the Rodnina group (Graf et al Nat Comm 2018) reported that L7/L12 interact with domain I to facilitate binding of RFs to the ribosome during termination. Surprisingly, this data is not even discussed despite being directly relevant to the story. Overall one gets the impression that the overall text is short and incomplete i.e. a Nature letter format and would benefit from being expanded to give credit to all the work related to the topic, rather than simply neglecting to even cite such works.

We thank Reviewer 1 for bringing up this important point. We have now added a paragraph before the Conclusion to discuss related work.

"In a recent paper on the role of RF3 in the dissociation of the release factors RF1 and RF2[40], the authors observed an interaction between domain I of RF1 and L7/L12 proteins, which assists the binding of RF1, as supported by complementary functional analysis using L7/L12 deletion mutants. However, such an interaction is not observed in our structures."

Reviewer #2 (Remarks to the Author):

The work presented in this paper is a great demonstration of the power of time-resolved Cryo-EM in structurally visualizing short-lived intermediates of biological processes. Here, it is used to resolve two conformational intermediates in the first 60ms of bacterial translation termination. The two intermediates were identified in structures of the 70S termination complex containing a P-site tripeptidyl tRNA, mRNA with UAA stop codon in the A site, and either RF1 or RF2 bound to the A site. The first intermediate (pre-accommodation state) shows the RF1/RF2 on the ribosome in the compact conformation similar to what is observed in solution ribosome-free or when bound to HemK methyltransferase. The second intermediate (accommodation state) shows the extended conformation of RF1/RF2 seen in previous crystal and Cryo-EM structures of termination complexes. This paper reports the first structural evidence of this conformational switch in the native pathway of translation termination.
The structural basis of stop codon recognition and peptide release in this work contributes greatly to our understanding of the high decoding accuracy in termination and that makes this work compelling for publication to the broader audience of Nature Communications. But to do justice to the work presented, there are a several places in this paper that require further clarification/elaboration/correction that would add strength to this story.

The larger changes that I request are below:
1. In page 2 line 46 it is written "Stop codon reading by RFs is aided by class-2 RFs…" Please clarify the differences in the roles of the bacterial (RF3) and eukaryotic/archaea (eRF3/aRF3) class-2 RFs.

We thank reviewer 2 for pointing this out. We now have added a few sentences to point out an important difference between RF3 and eRF3/aRF3 We note that eRF3, but not RF3, enters the ribosome in complex with GTP and eRF1(Alkalaeva et al, 2006), reminiscent of how the essential GTPases EF-Tu and eEF1 enter the ribosome in ternary complex with GTP and tRNA. From this analogy we speculate

that eRF3 enhances both efficiency and accuracy of stop codon reading. The main role of RF3 is in contrast to remove RF1/2 from the post-termination ribosome(Korostelev et al. 2010). RF3 action could therefore be made redundant just by reduction of the A-site affinity of RF1/2.

2. It is stated on page 3, line 112, that the Cryo-EM data show "somewhat faster conformational transition than in the quench-flow experiments". Please offer an explanation for why could be the case.

A possible explanation is that there might be a local temperature increase, due to the friction inside the microfluidic channel, which would accelerate the reaction.

We added a sentence in the main text to give an explanation to this point "The difference in termination rates is, we suggest, due to a local temperature increase by friction inside the microfluidic chip."

3. Are there any differences in the densities between the 24ms and 60ms datasets for the 70S-RF1 (extended) state?

The densities of the 70S-RF1 (extended) state are virtually identical (superimposable) from 24 ms and 60 ms, as confirmed by a correlation coefficient of 0.967.

4. After the phrase in page 4 line 128 "At a much later time-point" add in parentheses which specific dataset you are referring to (e.g. 45s data).

We thank Reviewer 2 for asking us to clarify this point. Here we are referring to the 45s dataset for RF1 and the 5h dataset for RF2. We changed the sentence to say "At a much later time-point (45s)".

5. Explain what was observed in the structure to conclude that the SCR motif is "bound loosely" (page 5 line 176), such as poor local resolution of these residues, etc.

We thank Reviewer 2 for pointing out this lack of clarity. In response, we added the alignment of SCR motif between compact and extended state to show the relative shift in Extended Data fig.4f. The comparison of the local resolution estimation of SCR motif is also shown in Extended Data fig. 4g and h.

6. Accompany the section of the paper talking about the recently published Bl-S-halted RF1 structure (lines 171-193) with a figure comparing the compact RF1 structure from this study with that from the Bl-S-halted complex.

We have followed this suggestion and aligned the BI-S halted RF1 structure with our compact RF1 structure in Extended Data fig.4b.

7. For Fig. 4, add reference to where the 200ms timepoint came from for the "peptide release RF-ribosome complex"

We thank Reviewer 2 for this suggestion. We had data from 200 ms timepoint in the previous version of the manuscript but in the current version it is not shown. Because of insufficient data collection and low resolution a firm conclusion regarding this point cannot be drawn. We have changed 200 ms to later time-point and we explain in the figure legend that the later time point is not known from our experiment, and we only know from another experiment that the final state was seen after 45s.

The smaller typographical changes are listed below:

1. Change "eRF1 and aRF1 factors read all –codons" to "eRF1 and aRF1 factors read all three stop codons" in page 2 line 46

2. Change "Gly-Gly-Glu triplet" to "Gly-Gly-Gln triplet" in page 2 line 50

3. Insert "of the bacterial 70S ribosome" after "70Å separating DC and PTC" in page 2 line 53

4. Add "Extended Data Fig. 5" to the figure reference "(Fig. 2e and 2f)" in page 4 line 128

5. Correct the "flipped-out nucleotides A1492 and A1913" in page 4 line 152 to "A1493 and A1913"

6. Add "Fig. 3b" to the figure reference "(Fig. 3d)" in page 4 line 152

7. Report the ratio of ethane-propane mixture in page 7 line 248
8. Replace "alkane" in page 8 line 256 with "ethane-propane mixture"
9. Correct the label in Fig. 3a and 3b from A530 to G530
10. Correct the x-axis of Extended Data Fig. 2b from "OH- (μM)" to "[OH-] (μM)"
11. Correct the Fig. 3d caption with "Gold: A1492 and A1493; and orange: S12"

We thank Reviewer 2 for pointing out the typographical mistakes. We have corrected them.

Reviewer #3 (Remarks to the Author):

Fu and co-authors describe their biochemical and cryo-EM work on 70S translation termination by RF1 and RF2. An unresolved question in the field is how RF1 and RF2 bind the ribosome transiently to probe an mRNA codon in the A site. The field in general agrees that RFs bind to the ribosome in a compact conformation and undergo a large-scale conformational change if a stop codon is recognized. But compact states have not been captured on the ribosome except in truncated mRNA-70S-ArfA-RF2 complexes and in a hyperaccurate RF1-70S complex in the presence of blasticidin S.

In this work, quench-flow experiments suggested a time course for the conformational change in a two-step reaction of peptide release. Time-resolved cryo-EM captured compact RF1 and RF2 at near-atomic resolutions of higher than 4 A. These structures provide insights into the early stages of codon sampling by RF1 and RF2, which involve stop-codon preorganization by RF1/RF2 for docking of the switch loop and subsequent insertion of the catalytic GGQ motif into the peptidyl transferase center. In summary, this manuscript describes important intermediates of translation termination.

I strongly recommend this manuscript for publication. I suggest the authors

address the following minor points:
1. Page 2, line #50. Correct "Gly-Gly-Glu" to "Gly-Gly-Gln".

We have corrected this mistake. We thank Reviewer 3 for catching it.

2. #62, a suggestion: change "GGQ-induction of ester bond hydrolysis" to "GGQ-mediated ester bond hydrolysis"

We have made this change.

3. #66-67, on SAXS. The authors mention their early SAXS work, in which they interpreted free RF1 as "extended in bulk solution". However other studies on RF1 and RF2 must be mentioned, which suggested a compact conformation of free RFs in solution:
a. SAXS (Zoldak et al; https://www.ncbi.nlm.nih.gov/pmc/articles/PMC1849895/)
b. FRET (Trappl and Joseph; https://www.ncbi.nlm.nih.gov/pubmed/26827724)

We added one sentence to cite the SAXS result from Zoldak et al. in the Introduction. "At the same time, SAXS data from T. thermophilus RF2 free in solution suggested a compact form for the factor or, possibly a mixture of compact and extended forms (Zoldak et al. 2007)". As for the FRET data from Trappl and Joseph, we mentioned their work in the manuscript multiple times.

4. Page, 3. #79 and all other instances of "Bl-S": replace "Bl-S" (which is confusing as it may be read as "B-one-S") with "BlaS". "Blasticidin S" is shortened to "BlaS" in numerous research papers, while "Bl-S" is not commonly used.

We agree with Reviewer 3, and we have changed all the Bl-S to BlaS.

5. While RF2 is discussed in text, no figures are shown with RF2 structures. Also,

it is hard to see how compact RF1 and RF2 are positioned on the ribosome relative to extended RF1 and RF2, respectively. To fill these gaps, add three (or more) figures/panels showing:

a. Alignment of compact and extended RF1 (aligned using 16S rRNA), in a view similar to figure panels 2c and 2d overlaid.

b. Alignment of compact and extended RF2 (aligned on 16S rRNA)

c. Alignment of compact conformations of RF2 in this work and in the ArfA-bound 70S complex (aligned on 16S rRNA).

In response, we have aligned the compact and extended RF1 and RF2 in similar view as in figure 2c and 2d and we added these figure panels in Extended Data fig.4.

REVIEWERS' COMMENTS:

Reviewer #1 (Remarks to the Author):

Interestingly, eRF3 is an essential protein while RF3 is not. We note that eRF3, but not RF3, enters the ribosome in complex with GTP and eRF114, reminiscent of how the essential GTPases EF-Tu and eEF1 enter the ribosome in ternary complex with GTP and tRNA. From this analogy we speculate that eRF3 enhances both efficiency and accuracy of stop codon reading. The main role of RF3 is in contrast to remove RF1/2 from the post-termination ribosome15. RF3 action could therefore be made redundant just by reduction of the A-site affinity of RF1/2.

The recent paper from Krebber and coworkers (doi: 10.1093/nar/gkz177.) suggests that eRF3 does not in fact delivery eRF1 as previously thought. Also it is unclear why to me the authors want to start to speculate about the role of eRF3 for stop codon accuracy in the introduction? The introduction should have facts and the speculations should be in the discussion. However, since this paper is about bacterial termination, perhaps it makes more sense for the authors to constrain their speculations for the topic of the paper?

the flipped-out nucleotides A1493 and A1913 (Fig. 3b and 3d).

A1913 is not in a helix so from where does it flip-out? This is certainly not clear from the Fig. 3b and 3d. Perhaps the authors meant A1492 not A1913?

Fig. 2 labels…

the convention is to write C74, C75 and A76 not 74C, 75C and 76A…!!

Second, at 60 ms, we observe the accommodated RF-ribosome complex with the extended form of RF and the tripeptide now in the exit tunnel and no longer attached to the P-site tRNA

The authors have managed to introduce many additional figures with densities and local resolution calculations but still do not managed to include in the paper a figure that illustrates that the authors have the resolution to distinguish between the tripeptide attached to the P-tRNA and hydrolysed…the current images are either too small and/or use segmented maps. The local resolution of 3.5A would not be sufficient to distinguish between the two scenarios. If the authors want to keep this interpretation then they should present evidence that they can distinguish a hydrolysed peptide from a non-hydrolyzed…zoomed images with non-segmented maps at different thresholds etc. Otherwise this interpretation should simply be removed from the manuscript.

on the identity of mono-peptide and di-peptide, with most found for the mono-peptide.

What are mono-peptides? My understanding was that a peptide was a short chain of amino acids…does one amino acid really count as a peptide? The authors might reconsider their nomenclature?

Reviewer #2 (Remarks to the Author):

I am satisfied with the response to all my requests and suggestions except for the first request regarding the text on class 2 RFs. Please correct the new citation added for the role of RF3 from "Korostelev et al. 2010" to "Freistroffer et al. 1997". Korostelev et al. 2010 article has no mention about the role of RF3.

I would like to add to the discussion by Reviewer 1 about the single-molecule data in Adio et al. 2018 that show a minor subpopulation of ribosomes found in the rotated state in the presence of RF1 and RF2. This contradicts not only with the CryoEM work in the manuscript under review, but also with previous single-molecule work from Puglisi and Gonzalez that showed that RF1 and RF2 stabilize the non-rotated state (Prabhakar et al. 2017; Sternberg et al. 2009). Prabhakar et al. 2017 also showed that the post-termination ribosome is in the rotated state when RF1/RF2 is not bound. This subpopulation may represent this post-termination ribosome unbound to RF1/RF2.

I approve this manuscript for acceptance.

Reviewer #3 (Remarks to the Author):

Previous reviewers' criticisms have been addressed in this revised version. A few minor points remaining to fix are:

1. Add full stop after the last sentence of the Abstract

2. In figure 4, the authors show 4 green filled circles to indicate a polypeptide. The caption says "green: tripeptide". It is not clear why the tripeptide is shown with 4 circles.

3. When superpositions of structures are shown, it is important to specify how the structures were superimposed. For example, in Ext. Fig. 4, differences between RF1/2 positions in different states are shown. If the superpositions were obtained by aligning RF1/2 structures they indicate structure differences/similarities. Alignment by 16S or by 23S rRNA would show positional differences within the ribosome.

4. Label the figure panels consistently with the capital or lower-case letters.

REVIEWERS' COMMENTS:

Reviewer #1 (Remarks to the Author):

Interestingly, eRF3 is an essential protein while RF3 is not. We note that eRF3, but not RF3, enters the ribosome in complex with GTP and eRF1 14, reminiscent of how the essential GTPases EF-Tu and eEF1 enter the ribosome in ternary complex with GTP and tRNA. From this analogy we speculate that eRF3 enhances both efficiency and accuracy of stop codon reading. The main role of RF3 is in contrast to remove RF1/2 from the post-termination ribosome15. RF3 action could therefore be made redundant just by reduction of the A-site affinity of RF1/2.

The recent paper from Krebber and coworkers (doi: 10.1093/nar/gkz177.) suggests that eRF3 does not in fact delivery eRF1 as previously thought. Also it is unclear why to me the authors want to start to speculate about the role of eRF3 for stop codon accuracy in the introduction? The introduction should have facts and the speculations should be in the discussion. However, since this paper is about bacterial termination, perhaps it makes more sense for the authors to constrain their speculations for the topic of the paper?

We have removed the speculation regarding eRF3 from the introduction part as reviewer #1 suggested.

the flipped-out nucleotides A1493 and A1913 (Fig. 3b and 3d).

A1913 is not in a helix so from where does it flip-out? This is certainly not clear from the Fig. 3b and 3d. Perhaps the authors meant A1492 not A1913?

The interaction is between the switch loop and the nucleotides A1493 and A1913. We have removed the wording "the flipped-out nucleotides" to avoid confusion.

Fig. 2 labels…

We have changed fig.2 labels to the same style as other figures.

the convention is to write C74, C75 and A76 not 74C, 75C and 76A…!!

We have changed the labels according to the convention.

Second, at 60 ms, we observe the accommodated RF-ribosome complex with the extended form of RF and the tripeptide now in the exit tunnel and no longer attached to the P-site tRNA

The authors have managed to introduce many additional figures with densities and local resolution calculations but still do not managed to include in the paper a figure that illustrates that the authors have the resolution to distinguish between the tripeptide attached to the P-tRNA and hydrolysed…the current images are either too small and/or use segmented maps. The local resolution of 3.5A would not be sufficient to distinguish between the two scenarios. If the authors want to keep this interpretation then they should present evidence that they can distinguish a hydrolysed peptide from a non-hydrolyzed…zoomed images with non-segmented maps at different thresholds etc. Otherwise this interpretation should simply be removed from the manuscript.

We have removed this interpretation from the manuscript.

on the identity of mono-peptide and di-peptide, with most found for the mono-peptide.

What are mono-peptides? My understanding was that a peptide was a short chain of amino acids…does one amino acid really count as a peptide? The authors might reconsider their nomenclature?

We addressed reviewer 1 concern on the off-pathway intermediates in the rebuttal by proposing the reason is likely the use of the di-peptide or one amino acid attached to the end of tRNA. Reviewer 2 brought up a better explanation for us to address this one. Therefore, we removed and modified the discussion on the rotated state of ribosome and mono-peptide or di-peptide attached to the tRNA. We believe the difference between Adio et al. 2018 paper and our manuscript is more possibly due to the subpopulation of post-termination ribosome unbound to RF1/RF2, as reviewer 2 suggested.

"The rotated-state subpopulation observed by Adio et al.[40] may represent the post-termination ribosome unbound to RF1/RF2, as also suggested by previous single-molecule work from Puglisi and Gonzalez labs[41,42]."

Reviewer #2 (Remarks to the Author):

I am satisfied with the response to all my requests and suggestions except for the first request regarding the text on class 2 RFs. Please correct the new citation added for the role of RF3 from "Korostelev et al. 2010" to "Freistroffer et al. 1997". Korostelev et al. 2010 article has no mention about the role of RF3.

We hope it is OK for us to remove the text regarding class 2 RFs from the introduction. As suggested by reviewer 1, class 2 RF3 is not the focus in this paper.

I would like to add to the discussion by Reviewer 1 about the single-molecule data in Adio et al. 2018 that show a minor subpopulation of ribosomes found in the rotated state in the presence of RF1 and RF2. This contradicts not only with the CryoEM work in the manuscript under review, but also with previous single-molecule work from Puglisi and Gonzalez that showed that RF1 and RF2 stabilize the non-rotated state (Prabhakar et al. 2017; Sternberg et al. 2009). Prabhakar et al. 2017 also showed that the post-termination ribosome is in the rotated state when RF1/RF2 is not bound. This subpopulation may represent this post-termination ribosome unbound to RF1/RF2.

We agree with reviewer 2 and we have modified our discussion on this point.

I approve this manuscript for acceptance.

Reviewer #3 (Remarks to the Author):

Previous reviewers' criticisms have been addressed in this revised version. A few minor points remaining to fix are:

1. Add full stop after the last sentence of the Abstract

We have modified the Abstract according to reviewer 3 and editor's suggestions.

2. In figure 4, the authors show 4 green filled circles to indicate a polypeptide. The caption says "green: tripeptide". It is not clear why the tripeptide is shown with 4 circles.

We have modified figure 4 to show only three circles to represent tripeptide.

3. When superpositions of structures are shown, it is important to specify how the structures were superimposed. For example, in Ext. Fig. 4, differences between RF1/2 positions in different states are shown. If the superpositions were obtained by aligning RF1/2 structures they indicate structure differences/similarities. Alignment by 16S or by 23S rRNA would show positional differences within the ribosome.

The alignments are done with 16S rRNA as reference in Ext. Fig. 4. We have added it in the legend of the Ext. Fig. 4.

4. Label the figure panels consistently with the capital or lower-case letters.

We have changed label for figure panels.
